# Acceptance of Technological Innovations in Emergency Departments: An Empirical Study Based on an Extended TAM

**DOI:** 10.3390/healthcare14101273

**Published:** 2026-05-08

**Authors:** Ann Thong Lee, R. Kanesaraj Ramasamy, Anusuyah Subbarao

**Affiliations:** 1 Faculty of Management, Multimedia University, Cyberjaya 63100, Malaysia; lee.ann.thong@student.mmu.edu.my (A.T.L.); anusuyah.subbarao@mmu.edu.my (A.S.); 2 Faculty of Computing Informatics, Multimedia University, Cyberjaya 63100, Malaysia

**Keywords:** technology adoption, influence factors, medical technology, emergency departments, ED, technology acceptance model, TAM, SPSS, SmartPLS

## Abstract

**Background:** Although technology is rapidly transforming many industries, the healthcare industry remains comparatively conservative and slow to adopt new technologies due to patient safety concerns. Notwithstanding the abundance of research on technology acceptance, most studies overlook departmental variations, making it impossible to enhance technology adoption in the medical sector. Thus, the purpose of this study is to bridge this gap by concentrating on the emergency department (ED). **Methods:** This study examined the factors influencing Malaysian ED healthcare professionals’ acceptance of new medical technology by introducing organisational support and training with the Technology Acceptance Model (TAM). The study’s target population comprised ED healthcare professionals in Malaysian hospitals who were at least 25 to 60 years old. In total, 140 valid surveys were gathered by email and WhatsApp from Malaysian hospital EDs, and SPSS and SmartPLS were utilised for analysis. **Results:** Perceived usefulness and training have a significant impact on attitude towards use, whereas attitude towards use is the sole variable that directly influences behavioural intention to use and acts as a mediator in certain paths. **Conclusions:** Hospital administration should concentrate on the actual needs of ED healthcare professionals, improve their understanding of technology, and offer targeted training in order to promote its effective adoption and utilisation. In the meantime, technology providers should improve the innovation’s design to make it more accessible to EDs. These findings also show that incorporating organisational support and training enhances TAM’s explanatory power and reveals its flexibility in high-stress, fast-paced environments.

## 1. Introduction

During the digital age, the rapid growth of technology has had a significant impact on nearly every aspect of society, as it contributes to the digital revolution in several industries globally [1]. In addition to bringing convenience and efficiency into daily lives, this period has also fundamentally changed the way traditional industries have always been [2]. Although many industries have benefited greatly from the widespread adoption of technology, the healthcare industry faces more difficult and particular challenges while implementing technology [3]. Considering the healthcare industry deals directly with life and health, each new technology presented must be thoroughly researched and reviewed. This is because it directly affects the patient’s life safety and overall medical quality, in addition to having a significant influence on routine medical procedures and healthcare professionals [4]. Therefore, the possible changes brought about by technology have caused the healthcare industry to be on edge and cautious [5].

In contrast to other industries, the use of technology in hospital settings must demonstrate not only that it can assist in increasing productivity and saving costs, but also that it does not put patients’ health and safety in danger. This extremely careful approach underscores the difficulties and challenges of extending technical innovation in this industry and reflects the high requirements of the healthcare industry for the use of technology. Consequently, there are still significant barriers and difficulties with the full implementation of new technology in the healthcare industry, even though technology has rapidly advanced and been widely used in other industries.

As of now, the healthcare industry has used a number of technologies, including electronic health records (EHR), to streamline information operations. This technology assists in preventing human mistakes and omissions while also increasing data processing efficiency and confidentiality [6,7]. Second, telemedicine technology reduces needless medical visits by using electronic programmes to give patients immediate monitoring and direction on home treatment [8]. Additionally, artificial intelligence (AI) is being utilised in the healthcare industry to evaluate vast volumes of medical data in order to increase the precision of healthcare professionals’ diagnoses and treatments [9].

Although there are new technologies available to help the healthcare industry progress, healthcare still primarily relies on labour and antiquated technology, since healthcare professionals are resistant to new technologies. Despite the fact that a great deal of studies have been done on the healthcare industry’s technology-related backwardness, the majority of these studies have not thoroughly categorised the different hospital areas. This has caused certain research findings to be inapplicable to certain specialised departments, including the emergency department. This is because different departments face different problems, and due to the particularity of each department, these problems cannot be fully expressed in other departments [10,11].

Studies on healthcare settings broadly frequently fall short of providing a workable solution to the unique issues facing every given department, and they do not allay the worries of healthcare professionals over the use of new technologies [12]. This restricts the potential benefits of technology and contributes to the slow growth in technology promotion in the healthcare industry. For instance, healthcare professionals in the emergency department might be more concerned about the dependability of technology in a high-stress setting to ensure it can be used swiftly and efficiently during emergency treatment. Meanwhile, the surgical department is more concerned about how technology will affect surgical accuracy [13]. Therefore, research that does not focus on a single aspect of the healthcare industry often falls short of offering effective suggestions and is unable to address the unique demands of different divisions owing to the differences in needs [12].

AI usage in general hospital departments is mostly concentrated on broader activities, according to Fahim et al. (2025) [14]. AI in these departments is mostly in charge of duties that do not directly involve patients, such as automating repetitive administrative procedures, predicting patient requirements, identifying diseases, and drug discovery [14,15]. However, Gün’s (2025) research on AI in the emergency department revealed that it is more likely to be used to directly support healthcare professionals in delivering quicker and more effective care [16]. For example, AI is utilised to provide clinical decision support specific to a patient’s condition, evaluate the severity of a patient’s symptoms in real time for triage choices, and so on. This implies that in order to provide prompt and precise clinical decision assistance in emergency departments, AI must function under time constraints and with sensitivity to the patient’s state [16]. These variations imply that compared to general departments, emergency departments have a harder time embracing AI. As a result, it is critical to specifically investigate technological acceptability in the emergency department context, rather than relying on studies from more stable clinical or administrative hospital settings.

Therefore, the TAM method is applied in this study as it is one of the most suitable methods for analysing technology acceptance. This is because a number of TAM-based studies have been carried out in hospital settings to hasten the adoption of medical technology in the healthcare industry. For instance, Alsyouf et al. (2023) found that the intention to utilise medical technology is positively correlated with perceived usefulness and perceived ease of use in a TAM-based study conducted in hospital settings [17]. Similar findings were also reported by Karkonasasi et al. (2023) [18]. Nevertheless, these studies lack emphasis on the particular requirements of each department, which contributes to a dearth of knowledge on the applicability and usefulness of TAM in hectic, high-pressure settings like the emergency department. This implies that by using TAM in this study, the deficiency of TAM in a fast-paced, high-pressure setting will also be explored.

In order to give a more thorough knowledge of technology acceptability, a number of studies have additionally taken into account external factors, including organisational support and training. For instance, Tetik et al. (2024) found that both organisational support and training significantly affect intention to use [19]. This suggests that these two are crucial in a medical environment. Nevertheless, there are few studies that take these two elements into account in a high-stress setting like an emergency department. Thus, this study aims to address the research void in the functionality of these two external factors in a high-pressure, fast-paced environment such as an emergency department by incorporating them.

It is believed that this study will fill the current research gap. From a theoretical perspective, this study extended TAM theory by introducing organisational support and training, thereby providing a more comprehensive understanding of technology acceptability in a hospital context, like the emergency room. Furthermore, the results of this study also have practical implications as emergency medical technology design can be improved to better suit a fast-paced environment, and the process of implementing technological advancements can be improved to make it more useful and realistic. In addition, focusing on the emergency department overcomes the lack of information on TAM’s suitability in hectic, high-stress environments while also offering a more thorough comprehension of technology adoption in these contexts.

In summary, this study focuses on the emergency department, with the goal of investigating the key factors influencing emergency department healthcare workers’ acceptance of new technologies, such as digital stethoscopes, smart devices, mobile applications, and so on, thereby effectively increasing the level of new technology application in the medical field under high-pressure conditions. Concurrently, this study seeks to offer pertinent perspectives, research objectives, and development for additional high-stress medical domains. These insights may provide strategies and opportunities for the digital transformation of health professional education and emergency department healthcare professionals worldwide by assisting educators and health administrators in redesigning healthcare professional training systems, organisational support structures, and pertinent professional learning models. These insights might also help medical policymakers reevaluate their existing approaches to introducing new technologies. To examine the reasons behind emergency department healthcare professionals’ resistance to medical technology, this study used the Technology Acceptance Model (TAM) as a theoretical framework that was modified to fit the study’s objectives. Specifically, this study’s primary focus was on three research questions:

1. What is the relationship between perceived usefulness, perceived ease of use, organisational support, training, and attitude towards use of new medical technologies by healthcare professionals in emergency departments?
2. What is the relationship between perceived usefulness, perceived ease of use, organisational support, training, and attitude towards use and behavioural intention to use new medical technologies by healthcare professionals in emergency departments?
3. What is the effect of attitude towards use on the relationship between perceived usefulness, perceived ease of use, organisational support, and training and the behavioural intention to use new medical technologies by healthcare professionals in emergency departments?

It is anticipated that this study will facilitate the seamless integration of modern technology in emergency departments, thereby enhancing the standard of medical treatment and lessening the strain on emergency department healthcare professionals. The results of the study could also have several benefits for stakeholders and further research. These benefits include providing medical policymakers with a foundation to build more effective regulations and support networks for the uptake of medical technology. The results can also be used by educators to create training programmes that better serve the requirements of emergency department healthcare professionals. Additionally, the findings of this study will provide technology developers with an idea to improve the design and usability of their products. In addition, it could also provide a foundation for further research into the factors affecting the uptake of medical technologies.

## 2. Literature Review

### 2.1. Technological Innovations in Emergency Departments

The emergency department has more responsibilities than other departments. Treating patients who need emergency rescue or who have sustained serious injuries is its first priority [20]. Due to the unique nature of the emergency department, emergency department healthcare professionals and resource management and coordination are important [21]. Emergency department healthcare professionals are required to allocate doctors, nurses, beds, and equipment in a timely and efficient manner while caring for patients that require emergency treatment [21]. Reasonable allocation is the secret to guaranteeing the effective operation of the emergency department and prompt patient response [21]. In addition to the above, the capacity of the emergency department to diagnose patients quickly is one of its key features. Emergency department healthcare professionals can quickly get comprehensive information about a patient’s condition using effective diagnostic techniques including imaging technologies and quick blood analysis, which strongly supports prompt treatment [21]. Additionally, the emergency department also places a high priority on patient admissions and transfers [22]. After considering and evaluating the condition of a patient, emergency department healthcare professionals thus decide whether to admit the patient into the hospital or organise a transfer for the patient into a different department for the needed treatment [22]. This technique improves the overall effectiveness of the use of medical resources while at the same time allowing the emergency department sufficient capacity for admitting new patients.

Together, these characteristics and approaches build an effective emergency department operational structure able to provide timely and quality care for ill or injured patients. Despite this, because of continuously increasing populations and medical needs, the integration of new medical technologies into emergency departments has grown more indispensable. This study referenced a number of studies and found that although some medical technologies have been gradually applied in emergency departments throughout Malaysia, their adoption and coverage are still insufficient compared to countries with highly developed medical technologies, such as the United States (US). In this regard, not only is the effective use of medical assets hindered, but also the emergency department’s ability to quickly and efficiently meet patients is limited.

Malaysia, as a developing nation with middle- and high-income levels, has implemented several innovative medical technologies in the emergency department [23]. For instance, some hospitals in Malaysia have adopted EHR systems, such as the MyEHR system, which facilitates the exchange of patient data [24,25]. Nevertheless, the coverage of this technology is restricted, and a single nationwide network has not yet been established [24]. It was found to have stability problems concurrently [26]. Rapid patient status changes in the hospital’s emergency department necessitate real-time data transfer for prompt clinical decision-making. The deployment of MyEHR may be further hampered by the emergency department’s inability to function efficiently due to erratic connection and system overload during peak hours.

Additionally, several Malaysian hospitals have also adopted telemedicine technology for facilitating patient support via online diagnosis platforms, prompted by the COVID-19 pandemic [27,28]. Nonetheless, the use of telemedicine in Malaysia is mostly restricted to simple consultation services with limited functionality [27]. In contrast to upper-income countries like the US, where telemedicine is highly developed and widely used in virtual healthcare applications, telemedicine in Malaysia has a limited application in emergency departments, as urgent treatment and on-site intervention are necessary, which also contributes to its limited use in these settings [27]. Furthermore, automatic external defibrillators (AEDs) have also gained inclusion within the Malaysian healthcare system [29]. Even so, the prevalence and usage of this technology in Malaysia fall significantly behind those of the upper-income countries, as only a few hospitals have placed it among emergency department services [29].

Furthermore, Malaysia has also embraced similar technologies, like the use of automated medication control systems, which have also been implemented in emergency departments of most hospitals, although the employment of AI-assisted diagnosis technology remains in its early stages [30]. This system allows emergency department healthcare professionals to improve the effectiveness of medicine distribution and reduce error rates [30]. Although the use of these technologies enhanced the emergency department’s operational efficiency, workforce shortages and insufficient medical information exchange persist in Malaysia. This is due to emergency department healthcare professionals’ lack of faith in these technologies, which leads to their refusal to use them. Additionally, although these technologies are now being integrated throughout the nation, only a small number of Malaysian hospitals have access to them. This implies that medical data cannot be fully connected to all hospitals nationwide. As a result, the adoption of new medical technology is proceeding slowly, and the healthcare business continues to struggle with a shortage of workers.

Nevertheless, it is thought that these new medical technologies can greatly enhance the healthcare delivery process, regardless of their being partially implemented, as is the case with MyEHR, telemedicine, and automated external defibrillators (AEDs), or still in the preparation stage, as in digital stethoscopes, smart devices, and mobile applications. Therefore, it is imperative that the implementation process be accelerated. Rather than just one particular medical technology, this study focuses generally across hospital emergency departments in order to get a more thorough picture of technology adoption. MyEHR, telemedicine, digital stethoscopes, automated external defibrillators (AEDs), smart gadgets, and mobile apps are some of the latest medical technologies that serve as examples for a deeper comprehension of the implementation problems.

In general, Malaysia still lags in the acceptance and usage of medical technology in the emergency department, which somewhat restricts the growth of medical service efficiency. Therefore, this study aims to explore the acceptance of medical technology in Malaysia from the perspective of healthcare professionals in emergency departments in order to mitigate existing problems and promote the further development of medical services.

Despite the fact that several studies have been carried out to enhance the adoption of medical technology, most of them fail to focus on particular departments and therefore cannot effectively address the problem of lagging medical technology application. Additionally, as most studies are based on other nations as the primary experimental locations, this may mean that the findings of these studies are unable to adequately address the issues Malaysia faces. This is because many nations deal with a variety of issues, such as disparities in resources and the environment. This emphasises the necessity of carrying out investigations that are customised for certain circumstances. Therefore, it is deemed vital to conduct this study, which focuses on Malaysia’s emergency department, and that is also seen to be able to compensate for the limitations of previous studies and present more useful recommendations and empirical results.

### 2.2. Theoretical Framework

Rapid scientific and technical advancements in recent years have resulted in the progressive integration of several cutting-edge technologies into many facets of human civilisation, improving people’s quality of life [31]. However, there is a lack of adoption of new technologies in some specialised industries, like healthcare, since people have concerns about the hidden risks and advantages of these technologies [32]. Many studies have been done to increase the use of technology, and one of the most concerned industries is healthcare. This is a result of the healthcare industry’s significantly lower adoption rate of new technology compared to other industries. Consequently, several studies have examined the factors influencing technology acceptability in an effort to boost the adoption rate of technology in the healthcare industry. The most frequently applied theoretical frameworks among these studies are the Technology Acceptance Model (TAM) and the Unified Theory of Acceptance and Use of Technology (UTAUT), demonstrating that they are applicable in this area [33].

Based on the Theory of Reasoned Action (TRA), Fred Davis introduced the Technology Acceptance Model (TAM) in 1989 to explain and forecast the adoption and application of technology [34,35]. Therefore, TAM is seen as an alternative to TRA and is also referred to as a simplified version of TRA. This is due to the fact that some TAM measuring methods are thought to be more suitable, accurate, and user-friendly than TRA in specific situations.

Venkatesh et al. (2003) proposed the Unified Theory of Acceptance and Use of Technology (UTAUT) in 2003 [36]. It incorporates eight current models, including TAM, to give a more complete framework for reaching more accurate findings [36,37]. According to Venkatesh et al. (2003) [36], UTAUT can account for as much as 70% of the variations in behavioural intentions [38,39].

#### 2.2.1. Comparison Between TAM and UTAUT

TAM is a theory which has been widely applied in numerous studies that have been conducted across different industries [17,40]. It has been used as a theoretical framework for numerous studies and has been found to be a suitable theory while conducting empirical studies [41]. Additionally, it has also been described as a method of simplifying the process of evaluating the use of technology [41]. The two most important constructs, PU and PEOU, give both the researcher and participants a deeper analysis of the technology adoption [17]. Furthermore, TAM is also flexible in that even when it is simplified, it can accommodate complex environments, such as healthcare, through other constructs besides those offered for the sake of the researcher [42]. However, there is also an argument that the simplicity of TAM is its flaw. It is argued that its simplicity shields the researcher from focusing on other aspects, which can constrain the explanatory and predictive capability of the model. Finally, while simplicity allows for convenient research, it may forgo some accuracy and richness of knowledge, which increases the danger of spurious inferences from research findings.

In comparison, UTAUT is a relatively recent theory that has already been applied in various studies in numerous industries [40,43,44]. According to the studies that have applied UTAUT, the theory provides a comprehensive framework that can be used in straightforward situations and manipulated in complex situations [45]. It encompasses both four fundamental constructs, PE, EE, SI and FC, and four moderator variables to facilitate the explanation of the acceptability of technology in its multifaceted nature and subsequently, on that basis, an enhanced knowledge of adoption factors [46,47,48]. However, sometimes, the multiplicity of these models can be a challenge. This is due to the fact that a complicated model will make the survey less flexible in certain situations and increase the time required for participation. Due to its complexity, its application in high-stress and time-sensitive settings such as the emergency department can be challenging. This is because the participants, such as healthcare professionals in the emergency department, may not be able to perform time-consuming assessments due to their busy schedules. Therefore, UTAUT is less appropriate for environments like the emergency department, where quick decisions are necessary and time is of the essence, because of its lengthy and complicated structure. This will therefore provide biased results.

Table 1 provides a thorough summary of the benefits and drawbacks of TAM and UTAUT. After comparing the two models, TAM was deemed more appropriate for this study. This is because the TAM without moderating variables is better suited to follow the objectives of this research, as moderator variables, such as age and gender, are not the focus of this study. Additionally, TAM’s simple and straightforward format makes it easier for stressed-out emergency department healthcare professionals to complete the survey quickly. As a result, although UTAUT is thought to have a more thorough explanatory capacity, its more complex structure makes it not suitable for time-sensitive settings. This demonstrates the importance of selecting a model that strikes a balance between explanatory power and practicality in hectic, high-stress environments like the emergency department. TAM is therefore more appropriate as the theoretical foundation for this study.

#### 2.2.2. Relevant Study Used TAM

This study references several relevant papers to demonstrate the reason why TAM is the most appropriate option for this study.

First of all, Walle et al. (2023) have conducted a study that used a modified version of the TAM to investigate healthcare professionals’ adoption intentions towards electronic personal health record (ePHR) systems under conditions of restricted resources [49]. In addition to analysing the two fundamental TAM variables of perceived usefulness and perceived ease of use, their study also added two additional variables to better assess healthcare professionals’ behavioural intention for ePHR systems in resource-constrained environments, which are information technology experience and digital literacy. Their findings demonstrated that medical practitioners’ adoption of the ePHR systems was significantly influenced by perceived usefulness, attitude, and perceived ease of use [49].

Second, TAM was also utilised by E. Y. Wang et al. (2023) to forecast paediatric healthcare professionals’ adoption of virtual reality technology [50]. In addition to confirming TAM’s efficacy in particular medical settings, their research also amended the TAM model by introducing a variety of demographic factors, such as age, gender, race, and clinical practice, in an effort to more accurately predict pediatric healthcare professionals’ behavioural intentions towards adopting virtual reality technology. According to their study, the propensity of healthcare practitioners to adopt and acquire the VR technology was highly impacted by the perceived usefulness, pleasure and the perceived ease of use. Meanwhile, the perceived ease of use for non-healthcare consumers was also affected by parameters like age, previous experience, cost, willingness to pay and curiosity [50].

Furthermore, Karkonasasi et al. (2023) also performed a study to investigate the acceptability of SMS vaccination recall and reminder systems in the Malaysian healthcare sector using a revised version of the TAM theory [18]. In order to accommodate a holistic exploration of all pertinent dimensions within this field, their research included additional variables, such as perceived compatibility and perceived concerns regarding privacy and security, within the framework of the TAM. The findings of their study demonstrated that the opinions of the nurses were positively influenced by the perceived system compatibility, the perceived usefulness, and the perceived ease of use [18].

Collectively, these studies show the adaptability and success of TAM in forecasting the adoption of the technology in the medical field. Despite the fact that these studies have shown the broad applicability of TAM in medical technology adoption research, the majority of them concentrate on general medical settings or more stable departments, such as paediatrics. This emphasis has prevented a complete verification of the explanatory capacity of TAM in high-pressure settings.

For example, the study conducted by Walle et al. (2023) [49] and E. Y. Wang et al. (2023) [50] extended TAM by including demographic variables, digital literacy, and IT experience. However, the participants in these studies were mainly from resource-constrained areas or particular groups, such as paediatricians and non-healthcare workers, which makes it challenging to represent the applicability of TAM in other clinical settings. In the meantime, although the study conducted by Karkonasasi et al. (2023) [18] was focused on Malaysia, the SMS vaccination reminder system they examined was basic and unable to capture the difficulties emergency departments have when using more sophisticated technology. As a result, comprehensive empirical data about the suitability of TAM in demanding and intricate medical settings is currently lacking. The results of this study, which therefore focuses on the emergency department, should fulfil this research gap and aid in evaluating the suitability of TAM in these high-pressure settings.

According to these studies, it is evident that TAM is suitable for this industry. The flexibility of the TAM model is further supported by the fact that most of these studies have added variables to TAM to increase its explanatory power. These findings indicate that perceived usefulness and perceived ease of use significantly influence the attitudes of healthcare professionals towards usage, as well as their behavioural intention to use. This highlights the significance of these two variables in the healthcare industry, emphasising their pivotal function in the adoption of technology in healthcare environments. However, there is a dearth of comprehensive examinations of the emergency department due to the fact that the majority of research has not focused solely on it. In order to increase the TAM model’s explanatory power and comprehend the emergency department’s priority for technological advancements, this study employed the TAM model with a focus on the emergency department.

Although there are some studies aimed at emergency departments, the majority of them are carried out in nations with environments that differ from Malaysia’s, such as China, the US, and others. This draws attention to a study gap on the adoption of technology in Malaysian emergency departments. Therefore, the TAM theory’s inclusion in this study can further develop and assess TAM’s performance in a high-pressure environment like the emergency department, as well as addressing the gap in Malaysia’s emergency departments, and ultimately result in recommendations or improvements for other nations with comparable environments to Malaysia.

To provide a clearer perspective and deeper comprehension, Table 2 provides an overview of all the papers that were referenced in this study and applied the TAM theory.

Since TAM theory offered the fundamental framework for this study, its dependent variable, behavioural intention to use, as well as its independent variables, perceived utility and perceived ease of use, are included. These two independent variables are included in this study because they have been shown to have a substantial impact on technology acceptability in studies pertaining to the healthcare industry. Furthermore, attitude towards use is seen to be one of the significant factors that may influence users’ behavioural intention to utilise technology. As a result, TAM, with attitude towards use as a mediating variable, serves as the theoretical framework for this study. However, organisational support and training are also included in this study, since it is believed that these are two significant factors influencing the adoption of technology. Although organisational support and training have been referred to as facilitating conditions (FC) in UTAUT’s theoretical framework, this study separates them into two independent factors to better understand their distinct roles in technology adoption. For example, training may be one-time and targeted at developing certain skills, whereas organisational support is comprehensive and long-term, depending on the company [51,52,53].

Aside from these factors, organisational support is intended to provide a positive climate to employees. At the same time, there is an intended improvement in technical skills of employees in relevant technical disciplines through training [51,52,53]. Additionally, organisational support is usually used to remove structural and emotional barriers, such as employee resistance to change. In contrast, training is mainly focused on addressing important omissions in the knowledge and skills of employees, such as knowledge of new advanced technology [51,52,53].

The separation of organisational support and training in healthcare technology adoption research has been validated and supported in several studies. For instance, this claim is supported by a study by Epizitone et al. (2023) [54]. It was noted in that study that organisational support positively affects the behavioural intention to use [54]. Hence, healthcare professionals will be more likely to accept and use the technology if they perceive an adequate level of organisational support in becoming used to the new medical technology. This assertion received further proof by another study:. positive and strong effects in impacting healthcare professionals’ behavioural intention to use were found to be delivered by organisational support in Hussain et al. (2025)’s study [55]. This implies that the behavioural intention of healthcare professionals to adopt new medical technologies depends on whether the organisation provides sufficient support in adopting and actively using the medical technology. There was some other research indicating the same results, where the organisational support was among the most frequently cited factors influencing the adoption of healthcare technologies. In numerous studies mentioned earlier in this review, organisational support was found to be significantly and positively associated with behavioural intention to use [55,56]. Overall, one could conclude that these studies collectively support the assertion that organisational support stands as an important construct of technology acceptance in the adoption of technology in the field of healthcare.

On the other hand, the significance of training as an independent variable was also established in various other studies. First, there was the healthcare-related study that was conducted by Falahati-Marvast et al. (2025) [57]. Their study included training as one of the independent variables. Through this study, they established that the behavioural intention to use new medical technologies may be significantly determined from the training [57]. The indication that may be derived here is that healthcare professionals who have undergone training are more likely to use new medical technology compared to those who had no training. A similar indication was portrayed in the study done by Ferreira et al. (2025), in which the training was determined as a significant positive contributor to behavioural intention to use [58]. This implies that the training offered through the organisation may lead to an increase in the confidence of healthcare professionals in medical technology, and this consequently results in a positive behavioural intention towards technology adoption. Furthermore, there have also emerged various other studies that established the significant positive relationship between training and behavioural intention to use [55,59]. The observations of these studies established the significance of training solely in encouraging the uptake of technology in the healthcare industry.

As such, it is vital to distinguish between organisational support and training. This is because this would enable researchers and practitioners to understand the distinct contributions of organisational support and training in technology adoption by controlling for organisational support and training uniquely. This will contribute to the development of more realistic solutions that will ensure the effective deployment of technology. Therefore, organisational support and training are incorporated into the TAM model for analysis.

## 3. Conceptual Framework and Hypotheses

### 3.1. Conceptual Framework

**Figure 1:**
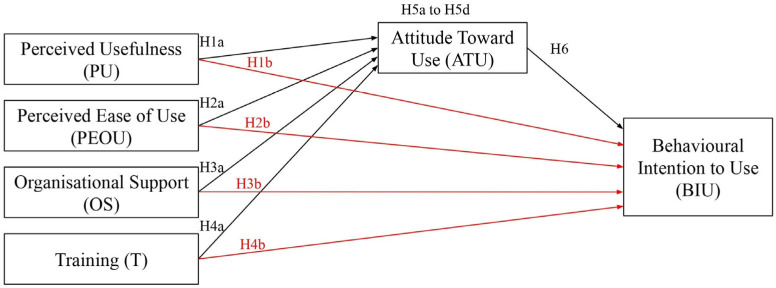
Revised TAM Framework for the Study.

The TAM model served as the theoretical foundation for this study, and the model was modified to fit its parameters, which included two variables, namely, organisational support and training. Figure 1 is provided to help illustrate the hypotheses in this study. All of the hypotheses of this study and the relationship paths between the variables are shown in detail in this figure.

According to Figure 1, the relationship paths for perceived usefulness, perceived ease of use, organisational support, and training in terms of attitude towards use are represented by H1a, H2a, H3a, and H4a, respectively. Furthermore, the relationship paths for perceived usefulness, perceived ease of use, organisational support, and training in terms of behavioural intention to use are represented by H1b, H2b, H3b, and H4b. Moreover, the mediating functions of attitude towards use between perceived usefulness, perceived ease of use, organisational support, training, and behavioural intention to use are represented by H5a through H5d.

### 3.2. Variables and Hypothesis Development

The theories that this study is going to examine will be discussed in this section. A total of 13 hypotheses were established after reviewing several past studies.

#### 3.2.1. Perceived Usefulness (PU)

“Perceived usefulness” (PU) refers to the extent to which a person thinks that employing a system might enhance productivity at work [17]. It is one of the main TAM theory components that has been brought up in a number of studies, and its significance in research on technology adoption has been emphasised.

Despite being a relatively new area of study, technology adoption in the healthcare industry has been extensively studied utilising TAM. Most of those studies highlighted how important the perceived usefulness is in influencing attitudes towards technology innovation in the healthcare industry [60]. The majority of studies indicated that healthcare professionals are likely to have a more positive attitude towards new medical technologies if they believe it would effectively decrease their burden and improve their job performance [50,61,62]. On the other hand, healthcare professionals may also have a positive behavioural intention to use it if they feel that new medical technologies make it possible to improve patient outcomes, streamline workflows, or increase decision-making accuracy [50].

Perceived usefulness is crucial in high-pressure and fast-paced settings like the emergency department. This is because emergency department healthcare professionals are required to handle emergencies, make rapid clinical decisions, and respond to sudden patient conditions. Therefore, technologies that can enhance decision-making and diagnostic precision are more likely to be adopted and utilised. Even if a medical technology is powerful, emergency department healthcare professionals may find it difficult to accept it if it does not meet the requirements of the emergency department. This led to the establishment of the following two hypotheses:

**H1a.**  *Perceived Usefulness has a positive relationship with Attitude Towards Use.*

**H1b.**  *Perceived Usefulness has a positive relationship with Behavioural Intention to Use.*

#### 3.2.2. Perceived Ease of Use (PEOU)

“Perceived ease of use” (PEOU) refers to the extent to which someone thinks using a specific system will require minimal effort [17]. It is an essential part of TAM in conjunction with perceived usefulness. Perceived ease of use has often been demonstrated to be a key factor affecting technology adoption in the context of the TAM.

With the advancement of technology in the healthcare industry, numerous studies have been conducted on the subject using the TAM model. The majority of the literature was developed based on perceived ease of use to determine its impact on medical technology adoption and professional behaviours in the healthcare industry. Most of those studies demonstrate evidence that healthcare professionals display a tendency to agree to having a positive attitude towards medical technology that was easy to comprehend and user-friendly. This finding has been replicated by several subsequent studies, which show that ease of use and shorter learning curves increase acceptance and usefulness, thereby leading to a positive attitude [49,50,61,62]. Additionally, there are several studies noting that the ease of use of technologies also impacted the intention of healthcare professionals to use technology [50].

Regarding the emergency department, it is crucial that medical technology is user-friendly for emergency department healthcare professionals. This is because emergency department healthcare professionals work in a hectic, high-stress setting where they are required to deal with a demanding workload and quickly shifting clinical situations under tight time constraints. As a result, they rarely have enough time to become proficient in using a complex medical technology. The clinical work process may be slowed down by a medical technology that is seen to require a lot of effort or attention, which might be a problem for emergency medical personnel. Therefore, medical technology that is simple, quick to master, and easy to use has a higher chance of being adopted and incorporated into routine emergency care procedures. This led to the formulation of the subsequent hypotheses:

**H2a.**  *Perceived Ease of Use has a positive relationship with Attitude Towards Use.*

**H2b.**  *Perceived Ease of Use has a positive relationship with Behavioural Intention to Use.*

#### 3.2.3. Organisational Support (OS)

Numerous studies, in addition to perceived usefulness and perceived ease of use, have also included organisational support (OS) as an important variable in extended TAM. Organisational support is defined as the degree to which employees feel that the organisation provides adequate resources and support for the technology to be effectively implemented [63,64,65].

The term “organisational support” also appeared in some studies in the health sector on technology acceptance, where it is identified as one of the important components of an emergent technology. There are several published studies on the topic that have suggested that if healthcare professionals receive adequate organisational support, such as technical support and resources available, they will develop a favourable attitude and eventually succeed in adapting and using a new system [19,66]. Additionally, several studies have shown that a high intention to use technology was attributed to well-organised support, highlighting the significance of the relationship between organisational support and the intention to use [67]. This support is instrumental in facilitating the process, reducing resistance to change, and increasing the likelihood of successful adoption of the technology.

Assistance from the organisation is crucial in the emergency department since the efficient use of medical technology greatly depends on usability of medical technology, fundamental technical support, and prompt technical support. Any interruption or delay in the operation of the medical technology might have a direct impact on patients’ outcomes and practical clinical decisions. As a result, strong organisational support could motivate emergency department healthcare professionals to embrace and use medical technology, such as a strong IT infrastructure, a responsive technical team, and managerial encouragement. Based on the above rationale, the following hypotheses are posited:

**H3a.**  *Organisational Support has a positive relationship with Attitude Towards Use.*

**H3b.**  *Organisational Support has a positive relationship with Behavioural Intention to Use.*

#### 3.2.4. Training (T)

In addition to support from the organisation, training has often been used in an attempt to explain technology uptake in TAM. The term “training” (T) refers to a structured approach for building someone’s capacity and knowledge with the intent of increasing productivity and effectiveness in the work setting [68].

Training has been found to provide the skills necessary for healthcare professionals to feel comfortable and competent in using a new technology within their respective healthcare contexts. Additionally, utilising a training approach can also mitigate fears about usability and resistance to adopting technology. There are a number of studies that have validated that appropriate training builds confidence and a more positive attitude towards adopting new technologies [69,70,71]. Furthermore, a few studies found that an established training model increased behaviour-based intention among healthcare professionals to use technologies, demonstrating a possible link between training and the behavioural intention to use [72,73].

Training is crucial in the emergency department because of the urgency and unpredictability of practical clinical practice. There is no space for trial and error, since emergency department healthcare professionals must quickly become proficient in medical technology to streamline their workflow. Without adequate and precise training, emergency department healthcare professionals may struggle to effectively utilise new medical technologies. This could have a negative impact on patient care and workflow. In contrast, a well-designed training programme can increase emergency department healthcare professionals’ familiarity with medical technology, reduce their fear of it, and ensure that they will be able to use it confidently in a practical clinical setting. As a result, training has been added as an independent variable in this study, and the following hypotheses were developed:

**H4a.**  *Training has a positive relationship with Attitude Towards Use.*

**H4b.**  *Training has a positive relationship with Behavioural Intention to Use.*

#### 3.2.5. Attitude Towards Use (ATU)

In addition to perceived usefulness and perceived ease of use, the “Attitude Towards Use” (ATU) is also one of the main constructs within TAM. In conjunction with perceived usefulness and perceived ease of use, attitude towards use is used to determine the behavioural intention of an individual in adopting a new technology. It refers to the generalised likes and dislikes of an individual in relation to a certain technology [60].

Furthermore, since AUT involves the personal attitudes and beliefs that a person possesses towards an object of technology, it can have the potential to significantly affect the decision of an individual to adopt and employ the technology. According to several previous studies, people who have positive attitudes about technology tend to use and adopt technologies that they find useful and beneficial [49,60,74,75]. Conversely, a negative attitude can make the individual less willing to adopt technology that they consider unattractive or inconvenient, which could hinder adoption.

In the emergency department, the ways in which emergency department healthcare professionals view the use of new medical technology are crucial. This is due to the fact that even if people think medical technology is practical and simple to use, their utilisation of it depends on how positively they feel about it. Because the emergency department is a high-risk and fast-paced setting, emergency department healthcare professionals typically employ the medical equipment that they are experienced and confident in using in a real-world clinic setting. Therefore, their readiness to adopt new medical technology in a real-world clinical setting may be greatly influenced by their attitude about its utilisation. Consequently, the following hypothesis was created:

**H6.**  *Attitude Towards Use has a positive relationship with Behavioural Intention to Use.*

#### 3.2.6. The Mediating Effect of Attitude Towards Use

In this study, “attitude towards use” (ATU) is also proposed as a mediator variable, which mediates the effect exerted by the independent variables on the dependent variable.

Numerous studies have been conducted, all demonstrating a positive relationship of several independent variables with attitude towards use, including perceived usefulness, perceived ease of use, organisational support, and training [49,50,61,62,71]. In addition, several studies have established that behavioural intention to utilise emerging technologies as a dependent variable is positively affected by attitude towards use [60,62,75]. Based on these findings, it is valid to propose that attitude towards use may be a mediator for the relationship between the independent variable and the dependent variable. Several studies conducted recently, including those by Ren and Zhou (2023) [76] and Walle et al. (2023) [49], have provided support for this perspective.

In the emergency department, attitudes on the use of new medical technologies are also significant mediator variables. This is due to the fact that emergency department healthcare professionals typically operate in high-stress, time-constrained, and unpredictable environments. Emergency department healthcare professionals are still reluctant to employ medical technology unless they have a favourable attitude towards it, even if it is believed to be helpful, simple to use, supported by an organisation, and has a well-structured training program. This is because medical technology has to operate in a high-stress setting, making faith in it crucial. Therefore, attitude towards use may be a psychological process that transforms perceived usefulness, perceived ease of use, organisational support, and training into strong behavioural intention to use. Accordingly, the subsequent hypotheses were put forth:

**H5a.**  *Attitude Towards Use positively mediates the relationship between Perceived Usefulness and Behavioural Intention to Use.*

**H5b.**  *Attitude Towards Use positively mediates the relationship between Perceived Ease of Use and Behavioural Intention to Use.*

**H5c.**  *Attitude Towards Use positively mediates the relationship between Organisational Support and Behavioural Intention to Use.*

**H5d.**  *Attitude Towards Use positively mediates the relationship between Training and Behavioural Intention to Use.*

## 4. Methodology

### 4.1. Survey Instrument and Data Collection

In order to guarantee compliance and safeguard participant privacy, this study underwent ethical approval prior to data collection. This study applied for ethics approval from Multimedia University’s Research Ethics Committee to highlight its ethical focus. The application was approved on 3 July 2024, with the approval number of EA0232024. This detail of the application is listed on the cover page of the study, along with the privacy and personal data protection act (PDPA) conditions. Additionally, participants were also given the assurance that their data would remain anonymous, as no personally identifiable information was gathered. All questions and associated answers were utilised only for academic purposes related to this study. Furthermore, the gathered data were securely archived in an aggregated form and used as a baseline reference for future evaluations, and wereaccessible only to the researcher and the supervisor.

The intention of this study is to determine the influences that can hinder emergency department healthcare professionals’ acceptance of new technologies. Therefore, the main requirement for sample recruitment was that participants must be healthcare professionals, namely emergency physicians, between the ages of 25 and 60, who are currently working in emergency departments. This ensured that all of the professionals engaged had sufficient experience working in emergency departments to be able to offer sincere and informed feedback based on their experience, in addition to aiding with accurate representation and generalisability.

Since this study examines the adoption of technology by emergency department healthcare professionals, it was necessary to ensure that the sample was confined to emergency department healthcare professionals. Thus, the judgmental sampling method, one of the non-probability sampling techniques, was employed in this study with the aim of having only participants with relevance. The efficient use of judgmental sampling in similar studies conducted by Singh et al. (2022) and Vidhya and Venkatesh (2024) further supports its effectiveness for use and application in this study [77,78]. These studies concluded that the use of judgmental sampling is an acceptable option for research studies that require getting information from respondents who are in the exact position required for the study or have the same experience to provide essential information on the issues being studied. Therefore, the sampling method used is valid and appropriate for the research aims of this study. Nevertheless, there are advantages and disadvantages to the judgmental sample approach. By using this approach, it can be ensured that every participant fulfils the requirements of the study because they have all operated with medical equipment in the emergency department. However, the approach also has disadvantages, such as the possibility of selection bias. This is because the participants are selected by the researcher according to the study’s requirements, rather than being chosen at random. As a result, the study’s sample was unable to fully capture all of the potential viewpoints in this field. Because the study solely focused on the hospital’s emergency department, its conclusions have limited applicability to other healthcare professions.

In addition, the sample size of this study has been determined using G*Power version 3.1.9.7, which is one of the power analysis tools that is frequently used in social science-related research. By using 5 predictors, a medium effect size of 0.15, a 5% margin of error, and a 95% statistical power, G*Power suggested a minimum sample size of 138 respondents. During the data collection stage, a total of 140 responses were gathered after an invitation email was issued to hospital emergency departments throughout Malaysia. One of the key metrics that is used to further support the sufficiency and dependability of survey data gathered for research is the response rate. However, it was not possible to obtain the entire quantity of survey distribution, since the survey was distributed by the hospital administration department after being received by the researcher. Therefore, the exact response rate for this study was not recorded. Nonetheless, the amount of the gathered data fulfils the minimal standard that is recommended by G*Power, which indicates that the collected data were adequate to facilitate a reliable statistical analysis and hypothesis testing.

Furthermore, a total of 2 data analysis tools were employed in this study during the data analysis process in order to obtain an accurate result. First, the “Statistical Package for the Social Sciences” (SPSS) version 28 was applied to examine the acquired data for outliers and then perform a descriptive analysis. This was to ensure the reliability of gathered data and thus the precision in results. The filtered data were then processed to carry out in-depth data analysis by using SmartPLS version 4.1.1.2 after the data analysis in SPSS. In this step, two distinct evaluations are involved, namely, measurement model evaluation and structural model evaluation [79]. Moreover, mediation analysis was utilised, as the attitude towards use was also a mediator variable in this instance. Finally, the Importance–Performance Map Analysis (IPMA) summarised the significance and the individual effect of all the variables.

### 4.2. Measurement Items

This study used the questionnaire survey technique and created a questionnaire to gather data. This is because the questionnaire survey technique is an easy and effective way to gather a lot of data, making it appropriate for quantitative research that needs a lot of data. Since this study uses a quantitative research approach, this approach was applicable. In addition, this study uses a cross-sectional research design, sometimes referred to as a one-time study. This approach satisfies the objectives of this study as it can quickly and easily gather the necessary data from a particular place.

A correlational method was used in this study to increase the accuracy of the research findings. This approach serves to lessen disruptions to the workplace and calls for less direct involvement from researchers. Additionally, the questionnaire was sent by email or WhatsApp in order to minimise in-person interactions and lessen the possibility of influencing respondents’ behaviour. Further guaranteeing the validity and dependability of the data was the fact that the study was carried out in the target hospital’s natural setting, which disrupted the normal tasks of the emergency department healthcare professionals little.

The questionnaire employed in this study consists of a cover page and 33 items separated into four sections, which are A, B, C, and D. The cover page provides a brief overview of the study’s history and includes an informed consent form to ensure that participants are aware of the study’s goals and willingly participate before completing the questionnaire. Three demographic questions, including years of work experience, gender, and age, are included in Part A. Additionally, Part B is broken down into four subsections, A, B, C, and D, for the four independent variables in this study, which are perceived usefulness, perceived ease of use, organisational support, and training. There are 5 items in each part, for a total of 20 items. Furthermore, Part C discusses the mediating variable, attitude towards usage, and includes 5 items. In addition, Part D concentrates on the dependent variable, behavioural intention to use, which also includes 5 items.

Table 3 provides a more thorough understanding of the questionnaire’s design structure by listing all of the variables and their corresponding items in this study. First, there are three items in Section A of the questionnaire, all of which are multiple-choice. Furthermore, Sections B, C, and D employed a 5-point Likert scale with 30 items in total. A rating scale of 1 to 5 was used to guide participants’ responses, where 1 represents “strongly disagree,” 2 represents “disagree,” 3 represents “neutral,” 4 represents “agree,” and 5 represents “strongly agree.” Participants are able to more correctly express their thoughts with the use of this rating approach.

The 5-point Likert scale was used in this study, since it is more straightforward and understandable than the 7-point scale. With just five choices, it is easier for responders to comprehend and reply rapidly, which is especially helpful for emergency department healthcare professionals, who are pressed for time [80,81]. Additionally, TAM-based medical research has made extensive use of this scale. Its ability to measure individual attitudes and opinions is therefore indirectly demonstrated [82,83,84].

Table 3 is provided in order to more clearly display the measurement techniques and associated data for each variable in this study. The variable name, code, section, number of items, and measurement technique are all listed in Table 3.

Additionally, Table 4 is included to better comprehend each of the items. All of the items are presented in Table 4, along with the reference from which they are derived, their respective codes and corresponding variables.

The items for perceived usefulness, perceived ease of use, attitude towards use, and behavioural intention to use are derived from studies by W. H. Cheah et al. (2023) [85] and Tu et al. (2022) [74], which are related to the healthcare industry but failed to specifically focus on the emergency department. However, items for organisational support and training that are derived from Islam and Hosen (2023) [86] and Nasongkhla and Shieh (2023) [87] are unrelated to the healthcare industry. As a result, these items have been modified in this study to better reflect the emergency department environment.

Because of the contextualisation and adaptation of those questions, a pre-test was conducted before the actual data collection for this study. This is to ensure the accuracy and applicability of the questionnaire, and thus, an accurate result may be obtained. Before the survey distribution, an expert in academic research was invited for assistance with the questionnaire evaluation process. The expert from the management faculty administered the pre-test for this study in August 2024, and the changes were made according to the comments. This pre-test enabled improvement in the format, phrasing, and clarity of the items, making the survey easier for participants to understand.

**Table 4:** Measurement Items and Sources of Constructs.

Variables	Reference	Code	Items
Perceived Usefulness (PU)	(Cheah et al., 2023) [85]	PU1	I feel using the new technology (Digital Stethoscope, Smart Device & Mobile Application) helps me perform my tasks in the emergency department effectively.
		PU2	I feel using the new technology (Digital Stethoscope, Smart Device & Mobile Application) boosts my productivity while working in the emergency department.
		PU3	I feel using the technology (Digital Stethoscope, Smart Device & Mobile Application) enhances my overall job effectiveness.
		PU4	I feel the technology (Digital Stethoscope, Smart Device & Mobile Application) simplifies my job duties.
		PU5	I feel my job performance improves when I use this technology (Digital Stethoscope, Smart Device & Mobile Application).
Perceived Ease of Use (PEOU)	(Cheah et al., 2023) [85]	PEOU1	I feel the technology (Digital Stethoscope, Smart Device & Mobile Application) is easy to use in my daily tasks.
		PEOU2	I feel the technology (Digital Stethoscope, Smart Device & Mobile Application) is clear and understandable.
		PEOU3	I feel I can quickly become skillful at using this technology (Digital Stethoscope, Smart Device & Mobile Application).
		PEOU4	I feel it is straightforward to get the technology (Digital Stethoscope, Smart Device & Mobile Application) to perform desired tasks.
		PEOU5	I feel learning to operate the technology (Digital Stethoscope, Smart Device & Mobile Application) is easy for me.
Organisational Support (OS)	(Nasongkhla & Shieh, 2023) [87]	OS1	I feel that my hospital’s leadership is committed to supporting the adoption of new medical technologies.
		OS2	I feel that my hospital provides adequate resources and infrastructure for using new technologies effectively.
		OS3	I feel that my hospital fosters an environment where new technologies are actively encouraged and promoted.
		OS4	I feel that technical support is readily available when I encounter issues with new medical technology.
		OS5	I feel that my hospital addresses and resolves challenges related to the implementation of new technologies.
Training (T)	(Islam & Hosen, 2023) [86]	T1	I feel that the training provided for new medical technologies (Digital Stethoscope, Smart Device & Mobile Application) meets my clinical needs.
		T2	I feel that the training I receive is sufficient to understand the practical applications of new technologies (Digital Stethoscope, Smart Device & Mobile Application).
		T3	I feel that I receive regular updates and additional training on new technologies (Digital Stethoscope, Smart Device & Mobile Application) relevant to my role.
		T4	I feel that the training sessions are well-structured and easy to follow.
		T5	I feel that the training enhances my confidence and ability to use new technologies (Digital Stethoscope, Smart Device & Mobile Application) effectively.
Attitude Towards Use (ATU)	(Tu et al., 2022) [74]	ATU1	I feel the potential benefits of using this technology (Digital Stethoscope, Smart Device & Mobile Application) in my medical practice to be significant.
		ATU2	I feel the technology (Digital Stethoscope, Smart Device & Mobile Application) easy to use in my daily work routines.
		ATU3	I feel that using this technology (Digital Stethoscope, Smart Device & Mobile Application) enhances my professional efficiency and effectiveness.
		ATU4	I feel that the technology (Digital Stethoscope, Smart Device & Mobile Application) aligns well with my current work practices.
		ATU5	I feel myself satisfied with the technology’s (Digital Stethoscope, Smart Device & Mobile Application) performance in my medical tasks.
Behavioural Intention to Use (BIU)	(Tu et al., 2022) [74]	BIU1	I feel myself likely to continue using this technology (Digital Stethoscope, Smart Device & Mobile Application) in my future medical practice.
		BIU2	I feel that I am committed to adopting this technology (Digital Stethoscope, Smart Device & Mobile Application) as a regular part of my workflow.
		BIU3	I feel that additional support and training would increase my likelihood of using this technology (Digital Stethoscope, Smart Device & Mobile Application).
		BIU4	I feel that the potential of the technology for improved patient outcomes influences my intention to use this technology (Digital Stethoscope, Smart Device & Mobile Application).
		BIU5	I feel that observing my colleagues successfully using this technology (Digital Stethoscope, Smart Device & Mobile Application) increases my intention to use it.

## 5. Results

### 5.1. Descriptive Analysis

#### 5.1.1. Outlier Checking

This study used an outlier test to prevent erroneous findings from in-depth analysis. The preliminary test results for the outlier checking are shown in Table 5.

Table 5 shows that the Mahalanobis distance has a maximum value of 23.861. Given that there are five independent variables in this study, the value should be less than 20.52 based on the chi-squared distribution’s critical value [88]. However, the maximum value of the Mahalanobis distance in this study is 23.681, which is much higher than the crucial threshold of 20.52. This suggests that the sample contains outliers. Therefore, the outliers had to be eliminated in order to guarantee the validity of the analysis results and the quality of the data.

Additionally, the Cook’s distance is also included in Table 5. This table shows that the Cook distance’s maximum value is 0.955, which is less than the widely accepted criterion of 1. This indicates that there are no unusual cases in the data gathered for this study.

This study retested the dataset after removing the outliers to ensure that no outliers were present. The analytical findings of the data following removal are displayed in Table 6.

According to the table, there are no outliers in the dataset. This is because the maximum value of the Mahalanobis distance is 7.834, which is significantly lower than the critical value of 20.52. In addition, the maximum value of the Cook’s distance decreased to 0.556, which is much lower than the threshold of 1, indicating that the dataset does not contain unusual cases.

#### 5.1.2. Demographic Analysis

This study established a demographic data analysis section to more thoroughly explain the respondents’ fundamental characteristics, including factors like gender, age, and years of employment. Table 7 displays the pertinent findings.

Regarding gender, women made up the majority of the 139 respondents, with 80 individuals, or 57.6%. Males comprised the remaining 59, or 42.4%.

In terms of age, the majority of respondents are in the 30 to 34 age range (*n* = 48, 34.5%). The second age group is the 35 to 39 age range (*n* = 33, 23.7%). The third age group is the 25 to 29 age range (*n* = 20, 14.4%). The fourth age group is the 40 to 44 age range (*n* = 14, 10.1%). The subsequent group the 50 to 54 age range (*n* = 11, 7.9%). Next is the 55 to 59 age range (*n* = 7, 5%). In addition, the last age group is the 45 to 49 age range (*n* = 6, 4.3%). However, there are no respondents over 60 years old in this study.

As for years of employment, the majority of respondents had fewer than 5 to 9 years of experience working in the emergency department (*n* = 55, 39.6%). The second category is 10 to 14 years of experience (*n* = 42, 30.2%). The group with 15 to 19 years of experience is the third highest category (*n* = 19, 13.7%), followed by 25 to 29 years of experience (*n* = 13, 9.4%), and then by those with 30 years of experience (*n* = 6, 4.3%). The lowest-ranked group is those with 20 to 24 years of experience (*n* = 4, 2.9%). However, none of the respondents in this study had more than 35 years of experience.

### 5.2. Measurement Model Assessment

#### 5.2.1. Indicator Reliability, Internal Consistency Reliability and Convergent Validity

Assessing the reliability of the indicators and internal consistency is the first stage in evaluating a measurement model. In this stage, the reliability of the indicator is examined by using the factor loading value. Hair et al. (2021) propose that an ideal factor loading value for each indicator is 0.708 or higher [79]. Nonetheless, a factor loading value of at least 0.7 is also considered acceptable [79]. These both show a substantial absolute contribution from the indicator.

Next, this stage also analyses the dependability of the internal consistency of the model. A total of two methods were carried out in this study to perform the reliability analysis for the internal consistency, namely, the Cronbach’s Alpha and composite reliability (CR). Given that the criterion for Cronbach’s Alpha is 0.7, a construct with a Cronbach’s Alpha value of 0.7 or more is regarded as implying that it has adequate internal consistency [89,90,91]. Meanwhile, the composite reliability (CR) criterion is 0.60, which means that a construct is deemed acceptable if its CR value is 0.6 or above, whereas values of 0.7 or higher imply strong internal reliability [79].

Convergent validity is evaluated after the measurement model’s reliability assessment during the evaluation process. In this stage, the AVE value is employed to assess the convergent validity of the model. According to the convergent validity criterion, the AVE value must be 0.5 or above in order to indicate a substantial connection among the specific construct and its related indicators [79].

The findings of both the reliability and the validity analysis of this study, which cover the indicator reliability, internal consistency reliability and convergent validity, are listed in Table 8.

According to the table, the lowest factor loading value in this study is OS1, which is 0.847. Therefore, the factor loading values for each of the indicators are above the 0.7 threshold, indicating considerable absolute contributions.

Additionally, all the composite reliability values are also presented in this table. According to the value, OS and T have the lowest composite reliability value, which is 0.952. The PEOU comes next, with a composite reliability rating of 0.953. Following this, the ATU and BIU had the next highest composite reliability values, at 0.963. Finally, PU had the highest composite reliability score, at 0.970. Based on the values, all constructs had composite reliability scores above 0.9, which indicates strong internal reliability. It also suggests that the indicators are strong at consistently measuring their construct. This result validates the reliability of the measurement model, confirming that the model had a low construct measure error, and it was appropriate to measure emergency department healthcare professionals’ views of technology use.

In addition, this study also provided Cronbach’s Alpha as one additional layer of support for the results of the reliability analysis. For Cronbach’s Alpha, OS and T had the lowest score of 0.938. PEOU had a score of 0.939, followed by ATU and BIU, both of which had values of 0.952, and PU had the highest value of 0.961. Based on the results, all constructs passed the thresholds for Cronbach’s Alpha, and every construct achieved a score well above the 0.7 threshold. This indicates that all constructs had sufficient internal consistency to reliably measure the intended constructs.

Furthermore, the table shows that OS and T had the lowest AVE value, at 0.80. The PEOU comes next, with an AVE rating of 0.804. The BIU, which has an AVE of 0.838, follows next. Additionally, the AVE of ATU was 0.840, which was the second-highest AVE among these constructs. The last construct, which is the PU, had the highest AVE value, with 0.864. Across all of the constructs, the lowest AVE was 0.8, which is above the standard for convergent validity of 0.5. This indicates that constructs in the research accounted for a minimum of 80% of the variance in the indicators. Cheung et al. (2024) stated that a value of more than 0.7 is ideal [92]. Therefore, the AVE values for all constructs in this study, which include PU, PEOU, OS, T, ATU, and BIU, were above 0.7, indicating that all were optimal in terms of this standard. As a result, it could be suggested that the constructs are likely to have sufficient convergent validity and that the items are appropriate indicators of each construct. The results provided evidence that the measurement items in the current study had fairly high consistency with the constructs they accounted for, and therefore may be considered appropriate indicators of technology adoption for healthcare professions. This also validates the questionnaire design and demonstrates that the questions are well consistent with the latent variables they measure, hence validating the model’s convergent validity.

#### 5.2.2. Discriminant Validity

Discriminant validity represents the last stage in the measurement model appraisal. To ensure that each construct in this model has a distinct value and does not conflict with the others, discriminant validity is employed [79]. A total of two different methods are used in this step to show that each construct is unique. These methods are the Heterotrait–Monotrait Ratio of Correlations (HTMT) and the Fornell–Larcker criterion.

##### Heterotrait–Monotrait Ratio of Correlations (HTMT)

HTMT is a well-known discriminant validity method that has been proven to be the most dependable approach available today. It has a threshold of 0.9, based on the rule of thumb of the HTMT. To fulfil the discriminant validity, each construct is required to have an HTMT value that is less than 0.90 [79]. Table 9 displays the outcomes of the HTMT.

The HTMT values for each of the constructs are listed in the table, which also shows that all of the HTMT values were acceptable for each of the constructs, as they were all below 0.9. This shows that each of the constructs was empirically distinct from the others, which shows evidence of having discriminant validity. This finding suggests that all of the constructs in this study were empirically distinct and enabled participants to differentiate each of the related but different constructs. This is an empirical finding that can support the theoretical basis of the measurement model and always confirms the model’s ability to accurately capture these numerous but related constructs in medical technology adoption.

##### Fornell–Larcker Criterion

As one of the commonly used discriminant validity techniques, the Fornell–Larcker criterion is used in this study to determine the construct uniqueness. Since discriminant validity was the most dependable method before to the development of HTMT, and most researchers still maintain this belief, this study used this criterion to further corroborate the findings of the discriminant validity. The criterion is that the diagonal values of each construct, which are the square root of its AVE, must surpass its correlated values with the other constructs [93]. Table 10 displays the discriminant validity findings using the Fornell–Larcker method.

AVE values for each construct are shown in the table. The results show that each of the constructs located diagonally possesses an AVE value higher than its correlation with other constructs. This indicates strong evidence that the data from this study meet the threshold for discriminant validity. This suggests that participants in this study were able to differentiate between constructs that are related yet conceptually separate, and that each construct examined in the study is its own, empirically distinct entity. This also fortifies the model’s theoretical framework and validates its suitability for studies pertaining to the healthcare industry.

### 5.3. Structural Model Assessment

#### 5.3.1. Collinearity Assessment

During this evaluation, the analysis begins with an assessment of collinearity. Hair Jr. et al. (2021) propose that the VIF is able to be utilised in the collinearity assessment process to ascertain collinearity issues between constructs [79]. In accordance with the rule of thumb for collinearity evaluation, the data will have a collinearity issue if the constructs’ VIF value is 5 or above [79,94]. All this study’s hypotheses, the relationship between the constructs, and the VIF value for each of the hypotheses are summarised in Table 11.

According to the results of the collinearity assessment, the relationship with the highest VIF value, 3.790, is between ATU and BIU. The second-highest VIF value, 3.279, is found in the relationship between PU and BIU. Next are the following relationships: T and BIU, with a value of 2.890; PEOU and BIU, with a value of 2.595; PEOU and ATU, with a value of 2.536; T and ATU, with a value of 2.534; PU and ATU, with a value of 2.171; OS and BIU, with a value of 2.000; and OS and ATU, with a value of 1.997. According to Table 11, all of the VIF values are less than 5, indicating that this study has no multicollinearity issues.

#### 5.3.2. Path Coefficient

The path coefficients come after the collinearity assessment which is used to evaluate the magnitude of the path relationship between the constructs by referencing the standard beta. The standard beta value falls within the range of negative one (−1) and positive one (+1), where +1 reflects a positive path relationship and −1 reflects a negative path relationship. A strong relationship is shown by path coefficient values around +1 or −1, whereas a weak relationship is indicated by values close to 0 [79]. This stage further incorporates the t-value, *p*-value, and confidence interval bias to determine the significance of the relationship [79]. The results of the hypothesis testing are displayed in Table 12.

The findings in Table 12 show that the standard β values for each of the other hypotheses are all positive, indicating a positive relationship direction, except for the influence of T on BIU (H4b). This is because the β value of H4b is the lowest among all the paths, which is −0.005. This indicates that the negative relationship is very weak because it is in the opposite direction and very close to 0. In contrast, the influence of OS on ATU (H3a) has a positive relationship, but its β value is only 0.026, which is the weakest among all positive paths. However, with a β value of up to 0.706, the influence of ATU on BIU (H6) exhibits the strongest positive relationship.

Additionally, t-values were employed in this study to assess the significance of the path. Following the critical value criterion, three paths were identified, with 1.6449 denoting a 5% significance level and 2.3263 denoting a 1% significance level. According to the critical value criterion, the t-values of the three paths of PU to ATU (H1a, t = 7.260), T to ATU (H4a, t = 3.269), and ATU to BIU (H6, t = 5.914) all exceeded two significant levels, which indicates that these paths are statistically significant. As a result, this study used a more stringent significance threshold, which is 1%, to guarantee the validity of the findings, and the related critical value was 2.3263.

Furthermore, the *p*-value was also examined in this study. As per the table, a total of three hypothesised connections are validated by the statistical significance. These are H1a (β = 0.541, t = 7.260, *p* < 0.01), H4a (β = 0.306, t = 3.269, *p* < 0.01), and H6 (β = 0.706, t = 5.914, *p* < 0.01), all of which have *p*-values less than 0.001. On the other hand, the *p*-values of H2a (β = 0.125, t = 1.518, *p* > 0.01), H3a (β = 0.026, t = 0.338, *p* > 0.01), H1b (β = 0.124, t = 1.023, *p* > 0.01), H2b (β = 0.036, t = 0.337, *p* > 0.01), and H3b (β = 0.037, t = 0.531, *p* > 0.01) are all more than 0.01. This indicates that these hypotheses are not significant in this study, suggesting that they are not supported.

Moreover, the reliability of the results is further verified by using confidence intervals bias. The table indicates that the importance of H1a (0.411, 0.655), H4a (0.162, 0.472), and H6 (0.486, 0.884) is further supported by the fact that their confidence intervals do not contain 0. The remaining paths, such as H2a (−0.019, 0.251), H3a (−0.081, 0.165), H1b (−0.065, 0.335), H2b (−0.133, 0.216), H3b (−0.062, 0.174), and H4b (−0.131, 0.112), all have confidence intervals that include 0, suggesting that these hypotheses are not statistically valid [95].

#### 5.3.3. Coefficient of Determination (R^2^)

The third stage in assessing the structural model is the coefficient of determination (R^2^). It is used to calculate the inner model’s explanatory strength using the R-squared. Hair Jr. et al. (2021) state that the R-squared value falls between 0 and 1, with 0.25 to 0.50 being considered weak, 0.50 to 0.75 being considered moderate, and 0.75 and above being considered substantial [79]. The R-squared findings are displayed in Table 13.

According to the table, ATU has an R-squared of 0.736, which means that PU, PEOU, OS and T explain 73.6% of its variance. In accordance with Hair Jr. et al. (2021), with an R-squared of 0.736, the ATU indicates a moderate model as it has passed the 0.5 criterion [79]. In contrast, BIU has an R-squared of 0.721, which means that PU, PEOU, OS, T and ATU explain 72.1% of its variance. According to Hair Jr. et al. (2021), the BIU has an R-squared of 0.721, which means it also indicates a moderate model as it has passed the 0.5 criterion [79].

#### 5.3.4. Effect Size (f^2^)

The evaluation then proceeds to the effect size (f^2^), which is the fourth stage. It is used to determine the degree to which the independent variables may explain the changes observed within the dependent variable [79]. Following the effect size rule of thumb that was proposed by Cohen (1988), the effect of each relationship may be categorised into three tiers based on its effect size value, where the value falling between 0.02 to 0.14 suggests a small impact size, 0.15 to 0.34 suggests a medium impact size, and 0.35 and higher suggests a big impact size [79,96]. The following table, Table 14, delivers a summary of the effect size measurements derived from the study.

The results reveal that the connection between PU and ATU, which is H1a, and the relationship between ATU and BIU, which is H6, have f-squared values of 0.510 and 0.473, respectively. Both of them exceeded the 0.35 criterion, which suggests that their impact sizes are big [79]. In the meantime, the f-squared values for the relationship between PEOU and ATU, which is H2a, and the relationship between T and ATU, which is H4a, are 0.023 and 0.140, respectively. Both were above the 0.02 threshold, indicating small impact sizes [79]. The remaining relationships had impact sizes below 0.02, including the relationship between OS and ATU, which is H3a, the relationship between PU and BIU, which is H1b, the relationship between PEOU and BIU, which is H2b, the relationship between OS and BIU, which is H3b, and the relationship between T and BIU, which is H4b. The sizes of all effects were 0.001, 0.017, 0.002, 0.002, and 0.000, respectively, indicating that none of them had an effect [79].

#### 5.3.5. PLSPredict

The PLSPredict is the last stage in the structural model assessment process. In addition to R^2^ for assessing in-sample explanatory power, PLSPredict is employed for assessing out-sample predictive power [79]. PLSPredict has two statistics-based prediction errors, which are “root-mean-square error” (RMSE) and “mean absolute error” (MAE) [79]. The results of the PLSPredict are displayed in Table 15.

Since all of the indices of ATU and BIU have positive Q^2^ predictors, as shown in Table 15, the RMSE is utilised in this study. The PLS-SEM RMSE values for all indicators of both ATU and BIU were lower than the LM RMSE values. This indicates that both of them possess a strong capacity for prediction [79].

### 5.4. Mediation Analysis

The mediation analysis is used since this study contains one mediator variable, which is the ATU. It seeks to determine the degree to which the association of the independent variables with the dependent variable is influenced by the mediating influence of the mediator variable [97]. According to the general rule of mediation analysis, there are three levels of mediation effect. When only the indirect effect of independent and dependent variables is significant, a full mediation effect is identified [79]. When the direct and indirect effects are both significant, the partial mediation effect is identified [79]. When neither the direct effects nor the indirect effects are substantial, no mediation effect is identified [79]. Table 16 lists the analysis results related to the mediating effect.

According to the table, the standardised beta coefficient (β) shows that all paths are positive, which indicates that there is a positive relationship between all of the variables. Based on the results, H5a has the strongest correlation (β = 0.382) among them, followed by H5d (β = 0.216). Meanwhile, the correlations of both the H5b (β = 0.088) and H5c (β = 0.018) are relatively weak as their β value is close to 0.

Moreover, the t-value analysis reveals that the t-values of H5a (t = 4.722) and H5d (t = 2.852) surpassed the crucial value of 2.3263, attaining a significant level of 1%. On the other hand, the t-values of H5b (t = 1.392) and H5c (t = 0.331) are below the threshold of 2.3263, suggesting that the indirect effects of these two paths are not statistically significant.

Simultaneously, this study integrated the *p*-value for further assessment. According to the table, H5a and H5d may be considered full mediation routes, since their direct effects are not significant, and their *p*-values are both less than 0.01 in the table, confirming their strong indirect effects. Conversely, H5b and H5c had *p*-values larger than 0.01, which are 0.082 and 0.370, respectively, suggesting that these two pathways lack statistical validity and do not support mediation relationships.

Furthermore, the aforementioned conclusion is supported by the findings of the confidence interval analysis. The confidence interval biases of both H5a (0.254, 0.519) and H5d (0.109, 0.363) do not include 0, suggesting that their indirect effects are consistent and dependable. On the other hand, the confidence interval biases of both H5b (−0.007, 0.201) and H5c (−0.058, 0.119) contained the 0 value, suggesting that their findings are not significant [95].

The findings of this study indicated that for emergency department healthcare professionals, their behavioural intention to use new medical technologies is not directly influenced by their perceptions of the technology’s utility or their training. However, both of these factors may indirectly positively influence behavioural intention to use by changing attitude towards using technology. This indicates that hospital administrators should first change their attitudes towards using technology to help improve emergency department healthcare professionals’ behavioural intention to use technology. For example, emergency department healthcare professionals could have formed a positive attitude towards technology and thereby had a strong behavioural intention if they had received training on how to use the technology relevant to their role. Additionally, hospital administrators can enhance emergency department healthcare professionals’ attitude towards technology and ultimately improve behavioural intention to use by demonstrating how technology can help emergency department healthcare professionals complete their daily tasks.

In contrast, BIU was not significantly impacted by PEOU or OS. According to this study’s findings, PEOU and OS did not directly or indirectly affect BIU through ATU. This is because neither PEOU nor OS was significantly associated with attitude. As a result, they had no significant influence on the behaviour of emergency department healthcare professionals in terms of embracing new medical technology in this study.

**Figure 2:**
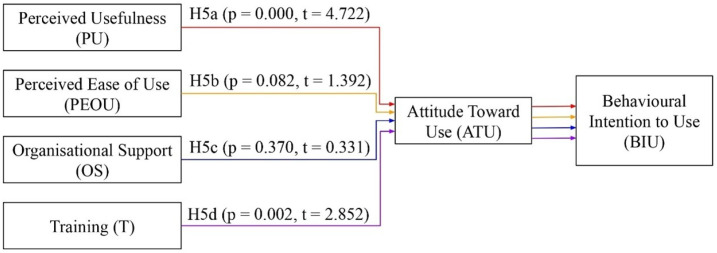
Mediation Analysis Model with Path Coefficients and Significance Levels.

To enhance comprehension of the mediation analysis, Figure 2 is presented. The figure displays every relationship with a distinct line colour, along with the corresponding t-value and *p*-value.

Figure 2 clearly demonstrates that the *p*-values for H5a and H5d are less than 0.01, suggesting that these two hypotheses are supported. However, the *p*-value for H5b and H5c is more than 0.01. This suggests that these two hypotheses are not supported.

### 5.5. Importance–Performance Map Analysis (IPMA)

**Figure 3:**
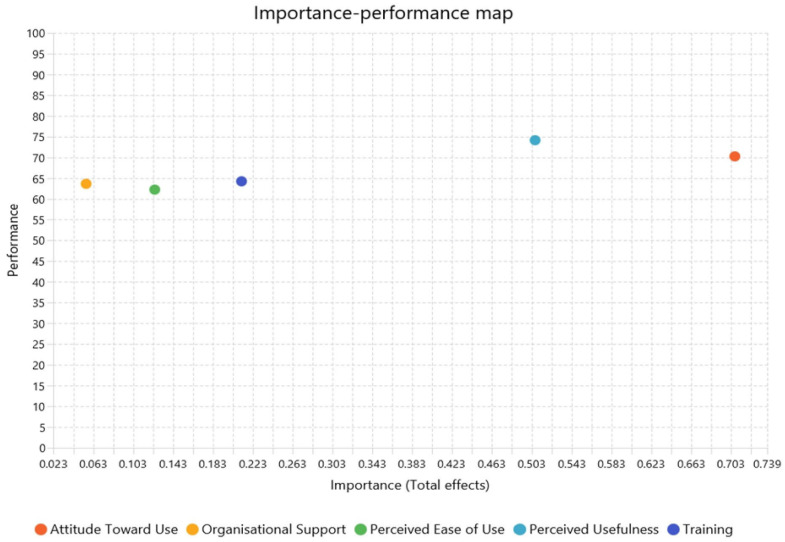
Importance–Performance Map.

The importance–performance map analysis (IPMA) is the final step in the process of data analysis. This analysis is used to measure the level of significance and performance of each construct to generate valid outcomes and insights [79]. The findings can be presented in two different formats, which are a figure and a table. In Figure 3, the constructs are represented in different colours to effectively present the result in a figure format. On the other hand, precise values of each of the constructs are displayed in Table 17.

Figure 3 uses “behavioural intention” as the dependent construct in the analysis. The placement of “attitude towards use” on the right side of the figure indicates it as the most significant and best-performing construct in the analysis. In contrast, “organisational support” is located on the left side of the figure, which suggests that “organisational support” has the weakest performance and importance among these constructs. The results indicate that researchers should focus on “attitude towards use” when looking to increase the usage of medical technologies in emergency departments, followed by “perceived usefulness”, “training”, “perceived ease of use” and “organisational support”.

The results of the IPMA with the precise values of every construct are displayed in Table 17. This table contains the study’s constructs, the importance value, which is also called the total effect, and the performance value.

As the table illustrates, the total effect of ATU is the largest, at 0.706. This suggests that the most important of these constructs is ATU. In addition, ATU has the second-highest performance score, which is 70.27, suggesting that it does an excellent job of boosting adoption intention. Additionally, the PU received a score of 0.506, the second-highest total effect value. This suggests that it is crucial for enhancing the motivation to adopt. Meanwhile, PU had the greatest performance score, which is 74.16, indicating that it performs the best in terms of enhancing adoption intention.

Conversely, OS has the lowest total effect value, which is 0.055. This suggests that, in comparison to other constructs, it has less impact on enhancing adoption intention. Next in line were PEOU, with a total effect value of 0.124, followed by T, with a total effect value of 0.211. On the other hand, PEOU has the lowest performance rating, with a performance value of 62.261. This suggests that when compared to other constructs, it performs the least well. OS, with a performance value of 63.662, and T, with a performance score of 64.267, come next.

Therefore, researchers and hospital administrators ought to have concentrated on both ATU and PU in order to increase the use of medical technology in emergency departments. This is due to the fact that they received the greatest performance value and total effect value.

According to the findings, hospital administrators should prioritise attitude, since it is the most crucial factor. The adoption rate of medical technology in emergency departments may also successfully increase. when enhancing the positive attitude of healthcare professionals. The positive attitude of emergency department healthcare professionals can be strengthened by providing in-depth information about the benefits and success cases of the technology. To achieve better results, hospital administrators should assign cases according to departments. For instance, hospital administrators might urge emergency department employees to share their good experiences with the technology, such as how it made them feel more supported, confident, or content with their jobs. Positive attitudes may be fostered by lowering staff worries and reinforcing positive thoughts through department-specific, accessible examples.

Simultaneously, attention should be paid to PU as the factor with the second-highest value in total effect and the highest performance value. By showcasing the advantages that technology may offer to particular departments, hospital administrators may effectively improve healthcare professionals’ perception of its usefulness in specialised areas like the emergency department. For instance, hospital administrators can share success stories from other emergency departments or host department briefings that highlight real performance metrics, like how well the technology reduces admission delays, to help make the advantages of the technology more concrete and compelling. When explicitly connecting the technology to better emergency department outcomes, emergency department healthcare professionals are more likely to think the technology is useful and worth using.

In addition, T is one of the factors that has to be considered, as it has the third-highest value for both performance and total effect. The total effect implies that it is an important factor in successfully enhancing the adoption of new medical technology by emergency department healthcare professionals. Nevertheless, it is evident from the performance value that the T was ineffective, which prevented a major increase in the use of the technology. Thus, hospital administrators should also reorganise training programmes to improve emergency department healthcare professionals’ use of technology. This can be accomplished through the use of simulation learning, which mimics real-life emergency department scenarios, the creation of short, modular training sessions that accommodate the shift schedule of a busy department, like the emergency department, and on-the-job training assistance from peer mentors or technology providers. Additionally, administrators should prioritise the system’s useful, task-oriented features over its overall capability when creating training so that emergency department healthcare professionals can use it with assurance in the hectic, time-sensitive environment of the emergency department.

On the other hand, although PEOU and OS had the lowest total effect and performance, hospital administrators may still increase overall emergency department healthcare professionals’ acceptance of technology by strengthening both of these factors. For example, hospital administrators should design user-friendly technologies with simpler interfaces, few navigation steps, and real-time advice for the high-stress emergency department setting in order to increase PEOU. In order to prevent integration issues and give emergency department healthcare professionals a period of time to become used to utilising technology, hospital management should evaluate the technology’s usability with emergency department employees prior to introducing it completely. In terms of OS, hospital administrators may demonstrate their backing for the deployment of technology by allocating healthcare professionals training sessions and incorporating emergency department healthcare professionals in pertinent decision- making. It is believed that by providing this kind of assistance, a positive atmosphere that promotes sustained usage may be established, boosting emergency department healthcare professionals’ confidence in implementing new technologies.

## 6. Discussion

### 6.1. Summary of Analysis

The findings of the hypothesis testing carried out in the previous chapter are summarised and presented in a table for easier comprehension in this section. Both the hypothesis and the decision are presented in Table 18.

This study examined the factors influencing Malaysian emergency department healthcare professionals’ adoption of technological improvements by using an extended technology acceptance model (TAM). Some noteworthy discoveries were obtained by adapting the structural model to the study’s parameters. The results of this study showed that emergency department healthcare professionals’ behavioural intention to utilise new medical technology is significantly influenced by their attitude towards use. Additionally, this study found that attitude towards use is the sole factor that can directly impact behavioural intention to use. Furthermore, the relationship between behavioural intention to use and perceived usefulness and training is found to be fully mediated by attitude. These findings suggest that emergency department healthcare professionals who have developed a positive attitude towards using new medical technology are more likely to adopt it. Consequently, this empirical model seems to have a considerable mediating impact, with attitude towards use acting as the primary mechanism between behavioural intention and external factors.

First of all, hypothesis H6 is supported by the findings of this study, that is, attitude towards use has a beneficial effect on behavioural intention to use consumption. According to the findings, it is noted that emergency department healthcare professionals holding positive attitudes towards medical technology has the potential to enhance their behavioural intention to employ the medical technology. This finding aligns with the core proposition of TAM, which also emphasises the importance of attitudes in determining behavioural intentions. At the same time, the results of this study are consistent with the results of several other studies. For example, Walle et al. (2023) discovered that the attitude of healthcare professionals had a big influence on their willingness to use medical technology [49]. Furthermore, Wang et al. (2023) noted that attitude has a significant role in encouraging intention to use [50]. This relationship was further supported by the study conducted by Gebeyew et al. (2025), which showed that healthcare professionals are more likely to exhibit a high propensity to employ medical technology when they perceive it has practical value, which may increase efficiency or lessen the burden [98]. In summary, the adoption by emergency department healthcare professionals of medical technology is significantly influenced by their attitude.

Furthermore, hypothesis H1a is also supported by one the study’s findings, which is that the perceived usefulness significantly improves a person’s attitude towards usage (ATU). This result is consistent with the original TAM model, since one of the primary constructs in the model is PU, which is thought to be effective in forecasting a user’s attitude towards adopting new medical technologies [99]. Additionally, this result is consistent with the studies that Gebeyew et al. (2025) [98] carried out. Based on their study, healthcare professionals are more likely to adopt medical technologies if technologies can help them be more productive in their daily duties [98]. A similar conclusion was reached by Luo et al. (2024), who found that attitudes towards the use of new medical technologies are strongly influenced by the PU [100]. According to their study, they thought that productivity, efficacy, and time efficiency, all of which were based on PU, had an impact on healthcare professionals’ positive attitudes towards new medical technologies [100].

Hypothesis H4a was also validated by this study, which revealed that training significantly influenced attitude towards use, by emphasising its function in forming positive user perception. Existing research also supports this result. For instance, Hussain et al. (2025) demonstrated nurses would be more likely to understand the importance of technology and rapidly master its primary functions if they had systematic, high-quality instruction [55]. Additionally, this training not only fosters a positive attitude and confidence towards the use of technology, but also offers emergency department healthcare professionals the opportunity to experience firsthand of how useful it is in clinical settings through hands-on demonstrations.

In addition to the direct path, this study contained indirect paths with attitude towards use as a mediating variable. This study reveals two important indirect relationships, where the relationship between perceived usefulness and behavioural intention to use (H5a) and the relationship between training and behavioural intention to use (H5d) are both fully mediated by attitude towards use. The support for H5a indicates that emergency department healthcare professionals are more likely to have a positive attitude and be more willing to use technology if they perceive that it will help them. This finding is consistent with the original theory of the TAM model and the results of studies conducted by Chawla and Joshi (2023) and Lee (2023), both of which demonstrate that perceived usefulness may indirectly impact behavioural intention by establishing pleasant attitudes [43,101]. Meanwhile, the support for H5d indicates that emergency department healthcare professionals who have received proper training are more likely to embrace technology because they have a more favourable outlook on it. This result is consistent with the findings of the study conducted by Dubale et al. (2024), which highlights the value of training in fostering adoption behaviour and enhancing attitudes [102].

Aside from the significant direct relationship between perceived usefulness, training, and attitude towards use, the hypotheses of H1b, H2a, H2b, H3a, H3b and H4b are not supported. This is because perceived ease of use and organisational support are shown to have no significant direct influence on attitude towards use, which H2a and H3a represent. In the meantime, perceived usefulness, perceived ease of use, organisational support, and training are shown to have no significant direct influence on behavioural intention to use, as represented by H1b, H2b, H3b, and H4b.

Nevertheless, the hypothesis of H1b is not supported due to perceived usefulness, which shows no discernible direct impact on behavioural intention to use (BIU). Although this is an unexpected result, it aligns with some previous studies that also studied the situation in a healthcare setting. This finding is substantiated through the studies by Walle et al. (2023) [49]. Walle et al. (2023) [49] conducted studies into predicting healthcare professionals’ intentions to use electronic personal health record systems. They discovered that perceived usefulness had no significant effects on healthcare professionals’ behavioural intention to use medical technologies [49]. This is due to the possibility that the introduction of new medical technologies may disrupt their work schedule and force them to modify their job routine, which may lead to a decrease in their effectiveness. However, time effectiveness is the top priority of the healthcare business due to its uniqueness, which may be one of the factors influencing emergency department healthcare professionals’ resistance to technology adoption. Accordingly, PU could not have a major impact on their behavioural intentions [103].

Furthermore, hypothesis H2a postulated perceived ease of use has no significant direct effect on attitude towards use. Some studies have come to a similar conclusion, despite the fact that this runs counter to the findings of the majority of studies [49,99]. Chau and Hu (2002), for instance, noted that perceived ease of use has a minimal impact on attitude towards use. Researchers believe that this may be related to the stronger professional ability of healthcare professionals, making it easier for them to adapt to complex systems, so this factor will not effectively affect the attitude of healthcare professionals in the emergency department towards new technologies [33]. In addition, Vanneste et al. (2013) also believed that unless ease of use is an indispensable condition, it will not significantly affect attitudes [33]. Emergency department healthcare professionals are more concerned about the practical value of technology than whether it is easy to operate, especially in high-pressure environments such as emergency departments.

Furthermore, hypothesis H2b in this study, which is that perceived ease of use has a positive relationship with behavioural intention to use, was also found to be not supported. Similar results were found by Gebeyew et al. (2025), who pointed out that perceived ease of use was not a decisive variable in nurses’ readiness to accept technology [98]. Nurses are more concerned with how technology may improve nursing quality than with how easy it is to use, particularly in high-stress settings like emergency departments, where the usefulness of the technology is the main factor. Thus, considering all of these findings, perceived ease of use is not necessarily a deciding factor in medical technology acceptance.

The next hypothesis is H3a, the effect of organisational support on attitude towards use, which was found to have no significant direct effect in this study. This runs against the findings of certain studies [104,105]. Nonetheless, a few studies have different opinions. For instance, Kwak et al. (2022) noted that some healthcare professionals are hesitant to change their work habits as the implementation of new technology may interfere with the current workflow [75]. Therefore, it is challenging to change emergency department healthcare professionals’ attitudes towards new technology, even with organisational support.

Next follows hypothesis H3b, the effect of organisational support on behavioural intention to use, which this study revealed to have no discernible direct impact. Despite the fact that this result is at odds with the findings of the majority of studies [67,104], some have come to similar conclusions. For example, Alsyouf et al. (2022) argue that if technology does not bring clear advantages, even if the organisation provides adequate support, healthcare professionals’ interest in and willingness to use technology will remain low [106]. It is evident that organisational support by itself is insufficient to encourage the use of technology unless such support can be converted into meaningful advantages for the workplace.

Additionally, hypothesis H4b was not validated because training did not significantly affect behavioural intention to use directly. This finding is aligned with the findings of some existing studies. For example, the study conducted by Xie et al. (2024) [107] discovered that healthcare professionals’ intention to use relevant medical equipment did not increase after training completion. This could be a result of their propensity to view training as an addition to theoretical information rather than as a means of imparting operational skills, particularly in clinical settings [107]. From this perspective, training lacks direct value for them, thus affecting their behavioural use intention of the technology. Additionally, it is extremely difficult to allocate more time for training due to the heavy workload of emergency department healthcare professionals, which may further weaken their trust and attention to training [107].

This implies that, although these variables are frequently highlighted in the adoption literature, their impact in an emergency department setting can be minimal or indirect. Thus, this study supports the view that in clinical settings, particularly emergency departments, actual application value, which is the key determinant of technology adoption, is more important than perceived ease of use and organisational support.

Moreover, hypothesis H5b was not validated, which means that attitude towards use failed to serve as a mediator variable between perceived ease of use and behavioural intention to use. This indicates that even if emergency department healthcare professionals have a favourable opinion of technology, which is caused by its ease of use, their desire to use it will not be greatly increased. Although this finding goes against the original TAM theory, it is in line with the findings of Siripipatthanakul et al.’s (2023) study, which noted that attitude towards usage is not always the key to influencing behavioural intention through perceived ease of use [108].

In addition, there was no evidence to support hypothesis H5c, which means that attitude towards use failed to function as a mediator between organisational support and behavioural intention to use. This suggests that, although the ease of use of the device may have elicited positive emotions among emergency department healthcare professionals, these attitudes did not increase intention to use the device. This finding differs from that of Malarvizhi et al. (2025) [109], but it is in line with Kwak et al. (2022), who argue that while organisational support contributes to promoting positive attitudes, its effect on actual intention to use is minor [75].

This study revealed several non-significant direct relationships when compared to the original TAM model, which indicates an important new discovery. Previous research highlights that behavioural intention to use TAM can be directly influenced by perceived usefulness and perceived ease of use. Nevertheless, this study shows no evidence of such a direct association. One possibility might be that the high workload and time sensitivity of the emergency department make emergency department healthcare professionals prioritise clinical relevance and actual value over organisational support or the simplicity of use of medical technology. Therefore, perceived ease of use and organisational support may not directly alter behavioural intention to use unless people have developed a favourable attitude towards utilising medical technology. This implies that attitudes regarding use have been crucial in converting external factors into behavioural intentions to use, which changes the TAM’s connection route in this context.

In conclusion, the results of this study demonstrated the critical role that attitude plays in improving emergency department healthcare professionals’ acceptance of new medical technology. Perceived usefulness and training can only impact behavioural intention to use when a positive attitude towards medical technology has been established. On the other hand, in this high-stress, fast-paced environment, the impact of perceived ease of use and organisational support is minimal. This may mean that the usage of medical technology in the emergency department is more dependent on whether emergency department healthcare professionals believe the technology is useful for their day-to-day clinical work than on its usability and support from the organisation. Therefore, hospitals should concentrate more on strategies that can improve emergency medical professionals’ attitudes towards the use of medical technology. For instance, hospitals ought to demonstrate the value of medical technology and offer practical training programmes to enable emergency medical professionals to quickly become proficient in it.

### 6.2. Theoretical Contributions

As technology has advanced, numerous studies have found the importance for the healthcare industry of adopting new developments, as technology is thought to greatly enhance healthcare procedures and service quality. However, the majority of the research concentrated on the overall hospital environment rather than the particular requirements of each department, which created a research gap in this field. Therefore, by starting with the emergency department, this study is expected to close the gaps in the current literature and offer theoretical and methodological references for further relevant research.

The TAM is used as the theoretical framework for this study. In order to capture possible influencing factors that impact the acceptability of technological improvements in the emergency department, this study incorporated organisational support and training into the TAM and redesigned it. This is due to the fact that prior research has shown that training and organisational support are both significant determinants of the factors that impact the adoption of technology advancements in the healthcare industry [1,67,69,70,71,73]. This extends the theoretical scope of TAM by including individual cognitive elements, organisational and environmental aspects, and so on.

According to the results, perceived ease of use and organisational support have no significant impact on the adoption of technology innovations in emergency departments. On the other hand, perceived usefulness and training are proven to have a considerable impact, which may be considered a key influencing factor in emergency departments. This result goes against the fundamental assumption of TAM that perceived ease of use is one of the most important factors that influence the behavioural intention. Based on the findings, emergency department healthcare professionals are more likely to base their decision to accept technology on usefulness than ease of use in a high-risk and fast-paced setting like the emergency department. This implies that the predictive ability of TAM could rely on the environment and could influence significant original TAM constructs, such as perceived ease of use. In addition to the above, this result demonstrates that including training can improve the theoretical explanatory power of TAM, as training is found to have a significant direct influence on attitude towards use and an indirect influence on behavioural intention to use. This implies that in high-pressure settings like the emergency department, TAM should be extended to incorporate external factors beyond personal perceptions in order to provide a more solid foundation for technology adoption. As a result, one of the theoretical contributions of this study is to provide an extended TAM model with a greater explanatory power in the medical profession, particularly in high-pressure settings like the emergency department.

The attitude towards use is one of the main components of the TAM model, although some researchers eliminated it from the framework because they thought that behavioural intention to use might be used to explain the attitude towards use, raising doubts about its theoretical validity. This research included attitude since it added two new factors based on the original TAM framework, which are organisational support and training. It was anticipated that training and organisational support would have an indirect impact on emergency department healthcare professionals’ intentions to utilise technology by influencing their attitudes. According to the results, attitude towards use still plays a mediating role in high-stress medical settings such as the emergency department. This is because the relationship between perceived usefulness and training and behavioural intention to use is strongly mediated by attitude towards use, offering further empirical evidence in favour of its theoretical validity. As a result, attitude towards use is a crucial mediator variable, particularly in high-stress medical settings like emergency departments, which is another theoretical contribution of this study.

In summary, this study offers important insights for particular improvements that must be made in the real use of technology in high-pressure settings by concentrating on the high-pressure setting of emergency departments. By extending the applicability of established models such as TAM to more specialised and demanding healthcare settings, these context-specific findings are able to provide information for the literature on technology adoption, which contributes theoretically. In this study, it was found that adding factors beyond subjective perception might considerably improve TAM’s explanatory power in hectic, high-pressure settings. In addition, this study demonstrated that attitude towards use is an indispensable mediating variable of TAM.

### 6.3. Practical Contributions

#### 6.3.1. Managerial Implications

Based on this study’s findings, technology suppliers and hospital administrators alike can benefit from the valuable suggestions that this study has provided. The purpose of the suggested recommendations is to increase emergency department healthcare professionals’ adoption and willingness to use technology advancements. Additionally, the suggestions are ranked according to the findings of the Importance–Performance Map Analysis (IPMA) in order to improve technology adoption more effectively.

In line with the Importance–Performance Map Analysis (IPMA), this study emphasises the significance of attitude towards use in affecting behavioural intention to use. According to IPMA, attitude towards use is the most important factor and scores second-highest in terms of performance. This finding reveals that emergency department healthcare professionals who have a positive attitude towards using medical technologies are more likely to have a favourable behavioural intention to use it. Therefore, hospital administrators should concentrate on encouraging emergency department healthcare professionals to have a positive attitude about the usage of new technologies. Hospital administrators should use useful tactics in addition to communications and meaningful statements to successfully improve emergency department healthcare professionals’ positive attitudes towards technology. For instance, hospital administrators should plan brief peer-sharing sessions during emergency department healthcare professionals’ breaks to emphasise prior successful experiences with technology adoption, given the hectic activity of the emergency department. Additionally, they can incorporate interactive technology demonstrations into their regular briefings. These approaches match the fast-paced nature of emergency department work while providing repeated, low-stress interactions that gradually increase emergency department healthcare professionals’ acceptability and confidence.

The results of this study also aid hospital administrators in allocating resources as efficiently as possible. The results imply that attitudes among emergency department healthcare professionals towards the use of medical technologies are influenced by perceived usefulness and ultimately affect their intention to use the technology. The IPMA results showed that perceived utility was effective in promoting the adoption of technology by the emergency department, ranking second in significance only to attitude towards use and performing best for behavioural intention. Hospital administrators should redirect resources for technology deployment in line with the findings in order to successfully improve the acceptance of technological developments by emergency department healthcare professionals. As a result, hospital administrators have to give top priority to funding emergency care-related technologies, such as technologies that provide smooth integration with critical systems like imaging, laboratory findings, and triage data, as well as real-time access to patient values. In addition, hospital administrators should solicit feedback from emergency department healthcare professionals before implementing the technology to verify that the technology can directly assist time-sensitive clinical procedures, hence increasing its perceived usefulness.

Additionally, this study also suggests that training can improve emergency department healthcare professionals’ views on the use of new technologies, which in turn influences their propensity to use new technologies in the future. Training rated third out of all variables in this study’s IPMA results, indicating that it significantly affects behavioural intention to use. Therefore, training not only serves to boost emergency department healthcare professionals’ technical confidence, but it is also a key factor in boosting technology adoption. Thus, hospital administrators should offer organised training programmes to enhance emergency department healthcare professionals’ technical competency based on this study’s findings. Hospital administrators must give training that is realistic and adaptable to the emergency department’s unpredictable and demanding environment. Consequently, it is recommended that hospital administrators create scenario-based training materials that replicate actual emergency department scenarios. This makes it possible for emergency department healthcare professionals to swiftly learn how to use technology. Additionally, hospital administrators can also provide emergency department healthcare professionals with access to brief educational materials, including short movies or app-based advice, which they may read on their phones during downtime.

It is anticipated that adopting these recommendations will result in a considerable rise in the acceptance and adoption of technological innovations by emergency department healthcare professionals. Patients may eventually gain indirect advantages from it. This is due to the fact that better workflows and the right technological assistance may greatly enhance emergency department healthcare professionals’ job performance and raise the standard of emergency treatment.

#### 6.3.2. Policy Implications

Numerous policy implications can be proposed based on this study’s findings. First of all, healthcare policymakers should concentrate on funding medical technology innovations and ensuring sufficient institutional support to address the emergency department’s heavy workload and workforce shortage. Meanwhile, increased investment in the healthcare industry may help acquire and improve the fundamental resources and infrastructure needed for medical technology, which leads to the effective adoption of new medical technologies. This can ensure system connectivity reliability, facilitate the seamless integration of electronic health record (EHR) systems, and offer sufficient technical support to facilitate real-time data access and effective operation in demanding clinical settings.

Moreover, a standardised training programme and a well-organised emergency medical technology guideline should also be developed by healthcare policymakers. This might assist emergency department healthcare professionals in being well-prepared for medical technology and rapidly becoming proficient in its actual application. In order to guarantee that all Malaysian hospitals have equal access to technology resources, healthcare policymakers should also balance the allocation of resources for each hospital. It is thought that by adhering to these initiatives, the long-term digital transformation of emergency medical services may be supported and the adoption and adoption of new medical technology might significantly rise.

#### 6.3.3. Implications for Technology Providers

The findings of this study also offer important implications for technology providers. This study found that healthcare professionals in emergency departments face particular technology challenges in a high-stress environment. In light of these challenges, technology providers should provide emergency departments with tailored solutions that are more practical and align with clinical practice. This might make it easier for healthcare professionals in emergency departments to adopt new technology. For instance, emergency department healthcare professionals should be involved in the creation of technology by technology providers so that usability testing may be done to handle the hectic and high-pressure nature of emergency care.

By doing this, the technology may be swiftly optimised for emergency departments to meet the objectives of increasing productivity and decreasing burden. For example, the technology can be used to expedite paperwork, provide notifications for patients whose conditions worsen and so on. Furthermore, technology providers may prevent issues in integrating new and existing systems, increase implementation speed, and prevent repetitive alterations that impact emergency department efficiency by consulting pertinent personnel, such as healthcare professionals. By implementing these tactics, technology providers are expected to significantly increase the application, acceptability, and general effectiveness of medical technologies in emergency department settings.

### 6.4. Limitations of the Research

Despite the fact that this work has made substantial theoretical and practical contributions, demonstrating its significance, there are several limitations that compromise the accuracy of the findings. First of all, this study used a quantitative research design and a cross-sectional research design for data collection. The cross-sectional study design is a method of data collection in which all the data are gathered at a single moment in time. By employing this methodology, this study may present an issue that findings cannot accurately represent the change in attitudes and behavioural intentions of emergency department healthcare professionals towards the use of medical technologies along with the time-to-time advancement of medical technology in emergency departments.

Second, the self-report approach employed in this study could have resulted in response bias. Response bias is the result of individuals giving socially acceptable replies, overestimating their level of technological proficiency, or completing the questionnaire carelessly or without fully comprehending the questions. Moreover, respondents were unable to communicate with the researchers directly because this study relied on Google Forms for data collection. This may have decreased the questionnaire’s overall dependability and affected the precision of the data gathered, since respondents who were having trouble with questions were unable to receive prompt assistance from researchers.

Additionally, the focus of this study is limited to four independent variables, which are perceived usefulness, perceived ease of use, organisational support and T. Hence, other potential influencing factors may be undervalued. Although this study concentrates on the important factors mentioned in earlier research, it is still possible that the model overlooked other factors that might impact the adoption of technological breakthroughs, such as peer pressure, system quality, or individual innovation capacity. The neglect of these factors might restrict the explanatory power of the model and limit the comprehensiveness of the research results.

Furthermore, the sample size of this study is 140, which is seen to be small in relation to the population of its intended respondents, even though the data came from a variety of Malaysian institutions. A larger sample size is ideal for obtaining reliable results when the population under research is large. Consequently, the results may not accurately reflect the perspective of emergency department healthcare professionals due to the small sample size of this study. This may eventually have resulted in inaccurate findings, such as false negatives, which are the failure to identify a real impact, or false positives, which are the detection of an effect that does not exist.

In addition, this study employed the judgmental sampling method, a non-probability sampling approach. Although this strategy allows researchers to include just the associated target group in the study, it has the downside of potentially decreasing sample representativeness and increasing the likelihood of selection bias. As a result, this study’s findings cannot accurately represent the viewpoints of all emergency department healthcare professionals.

Moreover, the study’s target group is limited to Malaysian hospital emergency departments, which has an impact on the results’ generalisability. This is due to the fact that diverse healthcare systems, hospital resources, and technological infrastructure may provide varied outcomes. As a result, the results of this study might not be entirely applicable to hospital emergency departments in other nations or healthcare environments.

### 6.5. Recommendations for Future Research

Following the discussion of the limitations of this study, several recommendations are presented for further research on the same topic. First of all, a longitudinal study design should be taken into consideration for future research. This method enables researchers to monitor the evolution of emergency department healthcare professionals’ attitudes and behavioural intentions from time to time along with the organisation’s assistance and training. This could result in more precise findings.

Furthermore, future research might consider conducting a study using a mixed-methods research approach. Because mixed-methods research designs incorporate both qualitative and quantitative research designs, researchers may use this design to conduct in-person interviews and engage with respondents while gathering survey data. As a result, researchers could ensure that only the respondents who fulfil the requirements of the research are selected. Meanwhile, researchers could also provide respondents with extra information so that they have a better understanding of the survey. This could effectively prevent respondents from filling out and submitting forms without as much deliberation or misunderstanding, which normally only results in inaccurate findings. Additionally, a mixed-methods study design would allow the researcher to observe the emotional responses of the respondents towards the use of new medical technology, which could provide further documentation of their attitude towards that technology.

In addition, future research could include additional variables to identify potentially influencing factors. The model of the theoretical framework of a research study, such as TAM, UTAUT, and so on, may have increased predictive power when additional variables are included. The increase in predictive power means that the model may have explanatory ability for the potentially influencing factors. This may ultimately build a greater understanding of the potential influencing factors that may affect the adoption of technology innovations. Thus, this may more likely result in a valid conclusion. For example, future research should include variables such as system quality, peer pressure, or previous technology experience, which may affect users’ attitudes and intentions. Hence, they will be able to develop a more complete model that reflects the real world better.

Future studies should also look at the approach in other high-stress settings, such as intensive care units. This is due to the fact that time constraints, uncertainty, and rapid decision-making are equally present in these circumstances. The results might thus be useful in determining how important attitude towards use is in impacting behavioural intention to use in various healthcare settings. This might aid in identifying the mediation impact of attitudes towards usage in various healthcare contexts.

Moreover, to better extend this research as a general estimation to other groups of healthcare professionals, a larger sample size should be examined. This is because a small sample size is a potential source of inaccuracies in the findings, whereas a larger sample size is better at creating a more accurate representation of the target population when the target population is larger. Furthermore, a larger sample size is simultaneously more advantageous for estimates and better able to identify the actual effect. On top of that, a larger sample size has the potential to support researchers in generalising findings to another situation. Thus, researchers should consider a larger sample size, as it allows an in-depth investigation of respondents’ interests, including age, employment function, years of experience, or hospital type, and their effects on behavioural intention. A large sample indicates a more heterogeneous sample size, as it includes more respondents and a range of their characteristics. This allows for greater richness of behaviour patterns that can inform the development of context-specific strategies for applying technologies for different groups of healthcare professionals.

## 7. Conclusions

The purpose of this research is to investigate why healthcare professionals in emergency departments are reluctant to adopt new technologies. In this research, the TAM model is used and is revised by adding two novel variables, which are “organisational support” and “training”. Since this study focused on emergency departments in particular, the target group is healthcare professionals working in emergency departments and aged from 25 to 60 years or older. The reasoning behind using professionals in this age range is that they would have demonstrable experience of working in an emergency department, which would provide a level of support or further insight into the use of medical technologies. In this study, Google Forms was used as the sampling tool to gather a total of 140 responses, and SPSS and SmartPLS were used for the analysis of the data collected.

Based on the results of this study, the variables “perceived usefulness” and “training” can be regarded as having an effect on the attitudes of emergency department healthcare professionals towards the use of medical technologies. Furthermore, the “attitude” of emergency department healthcare professionals towards the use of medical technologies has a significant effect on their “behavioural intention to use” them. Additionally, the relationship between “perceived usefulness” and “behavioural intention to use”, as well as between “training” and “behavioural intention to use”, is significantly mediated by the mediator variable, “attitude towards use”.

This study provides both theoretical and practical contributions based on the findings. From a theoretical standpoint, this study includes two variables in the TAM, which are “organisational support” and “training”. The extended TAM is expected to offer a more focused framework for studies on technology adoption in extremely demanding settings, such as the emergency department. Furthermore, this study demonstrated the necessity of the “attitude towards use”, highlighting the significance of including it as a mediator variable in high-stress settings such as the emergency department. This study has filled a research space in previous studies by revealing that attitude towards adopting new medical technology is important in the emergency department, since it is the only factor in this study that predicts behavioural intention to use. Additionally, it has been discovered that training and perceived usefulness might indirectly affect behavioural intention to use through the mediation of attitude towards use. In the meantime, behavioural intention to use is not significantly affected by perceived ease of use or organisational support. This may indicate that the adoption of new medical technologies in the emergency department does not entirely conform to the original path of the TAM model. The results of this study also indicated that the fast-paced, high-pressure environment of the emergency department may have an impact on the ways in which emergency department healthcare professionals respond to new medical technologies. Because of their unique circumstances, emergency department healthcare professionals are more concerned with cultivating a favourable attitude towards the use of medical technology before actually implementing it. As a result, the attitude towards usage appears to be more important in the emergency department compared to other settings.

From the practical standpoint, the results of this study offer helpful recommendations for both hospital administrators and technology providers to help them to optimise the process of implementing technology by focusing on factors that have a significant influence on the attitudes and behavioural intentions of emergency department healthcare professionals regarding the use of technology, such as “perceived usefulness”, “training” and “attitude towards use”. It is believed that future programmes could more effectively encourage the adoption of medical technology in emergency departments and better meet the actual needs of emergency department healthcare professionals by taking into account these important factors. In addition, this study also provides valuable perspectives for future healthcare-related research, such as employing longitudinal designs to document the changes in attitudes and behavioural intentions of healthcare professionals over time in order to enhance the accuracy of the findings and generate deeper insights. The research approach and findings of this study can serve as a guide to refine findings in other studies and carry out more thorough studies in the healthcare industry. Furthermore, the findings of this study might be investigated in other high-stress environments in the future. This might help to determine that the most important factor influencing behavioural intention to use is still attitude towards use. To better understand the mediation impact of attitude towards use in determining behavioural intention to use, future research might incorporate additional external characteristics, such as trust in technology, workload, and digital literacy, in addition to those included in this study. The findings of this study are expected to benefit all stakeholders, including hospital policymakers, hospital management, technology providers, and emergency department healthcare professionals.

In conclusion, this study can provide a more precise comprehension of the challenges that the emergency departments face during the adoption of new technologies by focusing on the unique conditions of a specific department, as well as how technological innovations can be successfully implemented into the emergency department setting. In this way, this study can successfully support a more seamless digital transformation in the healthcare industry. In addition to significantly enhancing hospital administration, this study found how perceived usefulness, training, and usage attitudes interacted. This result assists educators and policymakers in redesigning professional training curricula to better suit the emergency department setting, favourably impacting healthcare professionals’ attitudes towards technological learning, and contributing to global health professional education development. Furthermore, the findings of this study offer strategic direction for the implementation of novel technologies and useful recommendations to facilitate the efficient utilisation of medical technology in high-stress medical settings, such as emergency departments.

## Figures and Tables

**Table 1:** Comparison Table of TAM and UTAUT.

Methodology	TAM	UTAUT
Advantages	• Establishes a strong basis for empirical study. • Simplifies the difficult process of evaluating new technology adoption. • Uses two key constructs to facilitate rapid analysis and implementation. • Extremely flexible and adaptable.	• Provides a broad range of research topics with solid empirical support across industries. • Enables direct employment in a complicated setting by establishing a complex structure. • A thorough grasp of the influencing factors and a broad perspective on technology adoption are made possible by the complex structure.
Limitations	• May overlook factors that affect technology. • A reduction in explanation and prediction capacity may result from oversimplification.	• Survey design flexibility may be diminished by complex structures. • Time consumption may result in lower response rates from participants, particularly in busy or high-stress settings, such as emergency departments.

**Table 2:** Summary of Past Studies.

Authors	Research Objective	Variables	Outcomes	Research Gap
Walle et al. (2023) [49]	To examine the determinants influencing healthcare providers’ adoption of electronic personal health record (ePHR) systems.	• PU • PEOU • Information technology experience • Digital literacy • Attitude • Behavioural intention	• PU, PEOU, and attitude had a significant influence on the intentions of healthcare practitioners towards the adoption of ePHR.	• Sample mainly from resource-constrained areas, limiting generalisability to broader clinical settings.
E. Y. Wang et al. (2023) [50]	To determine whether the TAM model is reliable in predicting the elements that paediatric healthcare practitioners would consider when deciding whether to employ virtual reality (VR) as an anxiety reliever for hospitalised paediatric patients.	• PU • PEOU • Perceived enjoyment • Curiosity • ATU • Attitude towards purchasing • Age • Past use • Price willing to pay Attitude towards using VHC • Use and purchase of Virtual Reality (VR)	• Users’ willingness to use and purchase VR was significantly shaped by PU, PEOU, and pleasure. • The non-healthcare consumers’ PEOU was impacted by price, willingness to pay, age, past usage, and curiosity.	• Focus on specific participant groups, paediatric healthcare professionals, reduces applicability to emergency department settings.
Karkonasasi et al. (2023) [18]	To examine the factors that shape nurses’ attitudes and intentions in using Malaysia’s Virtual Health Connect (VHC) system for vaccination reminders via text messaging.	• PU • PEOU • Perceived compatibility • Perceived privacy and security • Attitude towards using VHC • Intention to use VHC	• Perceived system compatibility, PU and PEOU had a significant impact on the attitudes of nurses towards technology adoption.	• The technology studied is basic and does not reflect the complexity of emergency department technologies.

**Table 3:** Measurement of Variables and Questionnaire Structure.

Variable	Code	Section	Number of Item	Measure
Demographic Profile		A	3	Multiple-Choice
Perceived Usefulness (PU)	Independent Variables	B-Sub A	5	Likert 5-point Scale Strongly Disagree Disagree Neutral Agree Strongly Agree
Perceived Ease of Use (PEOU)	Independent Variables	B-Sub B	5	Likert 5-point Scale Strongly Disagree Disagree Neutral Agree Strongly Agree
Organisational Support (OS)	Independent Variables	B-Sub C	5	Likert 5-point Scale Strongly Disagree Disagree Neutral Agree Strongly Agree
Training (T)	Independent Variables	B-Sub D	5	Likert 5-point Scale Strongly Disagree Disagree Neutral Agree Strongly Agree
Attitude Towards Use (ATU)	Mediator Variable	C	5	Likert 5-point Scale Strongly Disagree Disagree Neutral Agree Strongly Agree
Behavioural Intention to Use (BIU)	Dependent Variable	D	5	Likert 5-point Scale Strongly Disagree Disagree Neutral Agree Strongly Agree

**Table 5:** Outlier Detection Results Before Removal.

Statistic	Minimum	Maximum	*n*
Mahal. Distance	0.035	23.861	140
Cook’s Distance	0.000	0.955	140

**Table 6:** Outlier Detection Results After Removal.

Statistic	Minimum	Maximum	*n*
Mahal. Distance	0.006	7.834	139
Cook’s Distance	0.000	0.556	139

**Table 7:** Demographic Summary.

Demographic Variable	Category	Frequency	Percent	Valid Percent	Cumulative Percent	Total
Gender	Female	80	57.6	57.6	57.6	139
	Male	59	42.4	42.4	100	
Age	25–29 years old	20	14.4	14.4	14.4	139
	30–34 years old	48	34.5	34.5	48.9	
	35–39 years old	33	23.7	23.7	72.7	
	40–44 years old	14	10.1	10.1	82.7	
	45–49 years old	6	4.3	4.3	87.1	
	50–54 years old	11	7.9	7.9	95	
	55–59 years old	7	5	5	100	
Years of Experience	5–9 years	55	39.6	39.6	39.6	139
	10–14 years	42	30.2	30.2	69.8	
	15–19 years	19	13.7	13.7	83.5	
	20–24 years	4	2.9	2.9	86.3	
	25–29 years	13	9.4	9.4	95.7	
	30–34 years	6	4.3	4.3	100	

**Table 8:** Reliability and Validity Results.

Construct	Indicator	Factor Loading	Cronbach Alpha	Composite Reliability	Average Variance Extracted (AVE)
Attitude Towards Use	ATU1	0.898	0.952	0.963	0.840
	ATU2	0.917			
	ATU3	0.933			
	ATU4	0.929			
	ATU5	0.905			
Behavioural Intention to Use	BIU1	0.894	0.952	0.963	0.838
	BIU2	0.932			
	BIU3	0.919			
	BIU4	0.928			
	BIU5	0.905			
Organisational Support	OS1	0.847	0.938	0.952	0.800
	OS2	0.895			
	OS3	0.922			
	OS4	0.890			
	OS5	0.916			
Perceived Ease of Use	PEOU1	0.890	0.939	0.953	0.804
	PEOU2	0.922			
	PEOU3	0.911			
	PEOU4	0.885			
	PEOU5	0.874			
Perceived Usefulness	PU1	0.919	0.961	0.970	0.864
	PU2	0.947			
	PU3	0.949			
	PU4	0.910			
	PU5	0.923			
Training	T1	0.897	0.938	0.952	0.800
	T2	0.868			
	T3	0.904			
	T4	0.906			
	T5	0.896			

**Table 9:** The Results of HTMT.

Construct	ATU	BIU	OS	PEOU	PU	T
ATU						
BIU	0.885					
OS	0.493	0.436				
PEOU	0.753	0.678	0.481			
PU	0.83	0.751	0.334	0.763		
T	0.708	0.607	0.746	0.641	0.526	

Note: 1. “Attitude Towards Use”—ATU; “Behavioural Intention to Use”—BIU; “Organisational Support”—OS; “Perceived Ease of Use”—PEOU; “Perceived Usefulness”—PU; “Training”—T; 2. Black background indicates same-variable comparisons with no results.

**Table 10:** The Results of Fornell–Larcker.

Construct	ATU	BIU	OS	PEOU	PU	T
ATU	0.916					
BIU	0.844	0.916				
OS	0.472	0.423	0.895			
PEOU	0.713	0.643	0.449	0.897		
PU	0.794	0.720	0.324	0.726	0.930	
T	0.672	0.579	0.702	0.601	0.504	0.895

Note: “Attitude Towards Use”—ATU; “Behavioural Intention to Use”—BIU; “Organisational Support”—OS; “Perceived Ease of Use”—PEOU; “Perceived Usefulness”—PU; “Training”—T.

**Table 11:** The Results of Collinearity Assessment.

Hypothesis	Relationship	VIF
H1a	PU -> ATU	2.171
H2a	PEOU -> ATU	2.536
H3a	OS -> ATU	1.997
H4a	T -> ATU	2.534
H1b	PU -> BIU	3.279
H2b	PEOU -> BIU	2.595
H3b	OS -> BIU	2.000
H4b	T -> BIU	2.890
H6	ATU -> BIU	3.790

Note: “Attitude Towards Use”—ATU; “Behavioural Intention to Use”—BIU; “Organisational Support”—OS; “Perceived Ease of Use”—PEOU; “Perceived Usefulness”—PU; “Training”—T.

**Table 12:** Hypothesis Testing Result.

						Confidence Intervals Bias		
Hypothesis	Relationship	Standard Beta	Standard Error	t-Value	*p*-Value	5%	95%	Decision
H1a	PU -> ATU	0.541	0.074	7.260 **	0.000	0.411	0.655	Supported
H2a	PEOU -> ATU	0.125	0.082	1.518	0.065	−0.019	0.251	Not Supported
H3a	OS -> ATU	0.026	0.075	0.338	0.368	−0.081	0.165	Not Supported
H4a	T -> ATU	0.306	0.094	3.269 **	0.001	0.162	0.472	Supported
H1b	PU -> BIU	0.124	0.121	1.023	0.153	−0.065	0.335	Not Supported
H2b	PEOU -> BIU	0.036	0.106	0.337	0.368	−0.133	0.216	Not Supported
H3b	OS -> BIU	0.037	0.070	0.531	0.298	−0.062	0.174	Not Supported
H4b	T -> BIU	−0.005	0.074	0.070	0.472	−0.131	0.112	Not Supported
H6	ATU -> BIU	0.706	0.119	5.914 **	0.000	0.486	0.884	Supported

Note: 1. ** significant at *p*-value < 0.01; 2. “Attitude Towards Use”—ATU; “Behavioural Intention to Use”—BIU; “Organisational Support”—OS; “Perceived Ease of Use”—PEOU; “Perceived Usefulness”—PU; “Training”—T.

**Table 13:** The Coefficient of Determination (R^2^) Results.

Construct	R-Square (R^2^)	R-Square Adjusted
Attitude Towards Use	0.736	0.728
Behavioural Intention to Use	0.721	0.711

**Table 14:** The Results of Effect Size (f^2^).

Hypothesis	Relationship	f-Square (f^2^)	Impact
H1a	PU -> ATU	0.510	Big
H2a	PEOU -> ATU	0.023	Small
H3a	OS -> ATU	0.001	No
H4a	T -> ATU	0.140	Small
H1b	PU -> BIU	0.017	No
H2b	PEOU -> BIU	0.002	No
H3b	OS -> BIU	0.002	No
H4b	T -> BIU	0.000	No
H6	ATU -> BIU	0.473	Big

Note: “Attitude Towards Use”—ATU; “Behavioural Intention to Use”—BIU; “Organisational Support”—OS; “Perceived Ease of Use”—PEOU; “Perceived Usefulness”—PU; “Training”—T.

**Table 15:** The Results of Effect Size (f^2^).

Indicator	Q^2^ Predict	PLS-SEM_RMSE	LM_RMSE	(PLS-SEM RMSE)-(LM_RMSE)	Remark
ATU1	0.541	0.510	0.570	−0.060	High Predictive Power
ATU2	0.622	0.522	0.585	−0.063	
ATU3	0.620	0.489	0.534	−0.045	
ATU4	0.588	0.516	0.593	−0.077	
ATU5	0.596	0.526	0.586	−0.060	
BIU1	0.540	0.528	0.590	−0.062	High Predictive Power
BIU2	0.494	0.525	0.583	−0.058	
BIU3	0.419	0.574	0.625	−0.051	
BIU4	0.474	0.543	0.564	−0.021	
BIU5	0.382	0.578	0.616	−0.038	

**Table 16:** The Results of Mediation Analysis.

						Confidence Intervals Bias			
Hypothesis	Relationship	Standard Beta	Standard Error	t-Value	*p*-Value	5%	95%	Decision	Types of Mediation
H5a	PU -> ATU -> BIU	0.382	0.081	4.722 **	0.000	0.254	0.519	Supported	Full Mediation
H5b	PEOU -> ATU -> BIU	0.088	0.063	1.392	0.082	−0.007	0.201	Not Supported	No Mediation
H5c	OS -> ATU -> BIU	0.018	0.054	0.331	0.370	−0.058	0.119	Not Supported	No Mediation
H5d	T -> ATU -> BIU	0.216	0.076	2.852 **	0.002	0.109	0.363	Supported	Full Mediation

Note: 1. ** significant at *p*-value < 0.01; 2. “Attitude Towards Use”—ATU; “Behavioural Intention to Use”—BIU; “Organisational Support”—OS; “Perceived Ease of Use”—PEOU; “Perceived Usefulness”—PU; “Training”—T.

**Table 17:** The Results of IPMA.

Construct	Importance (Total Effect)	Performance
ATU	0.706	70.27
OS	0.055	63.662
PEOU	0.124	62.261
PU	0.506	74.16
T	0.211	64.267

Note: “Attitude Towards Use”—ATU; “Organisational Support”—OS; “Perceived Ease of Use”—PEOU; “Perceived Usefulness”—PU; “Training”—T.

**Table 18:** Summary of Hypothesis Test Results.

Hypothesis		Decision
H1a	Perceived Usefulness has a positive relationship with Attitude towards Use.	Supported
H2a	Perceived Ease of Use has a positive relationship with Attitude towards Use.	Not Supported
H3a	Organisational Support has a positive relationship with Attitude towards Use.	Not Supported
H4a	Training has a positive relationship with Attitude towards Use.	Supported
H1b	Perceived Usefulness has a positive relationship with Behavioural Intention to Use.	Not Supported
H2b	Perceived Ease of Use has a positive relationship with Behavioural Intention to Use.	Not Supported
H3b	Organisational Support has a positive relationship with Behavioural Intention to Use.	Not Supported
H4b	Training has a positive relationship with Behavioural Intention to Use.	Not Supported
H6	Attitude towards Use has a positive relationship with Behavioural Intention to Use.	Supported
**The Mediating Effect of Attitude Towards Use**		
H5a	Attitude towards Use positively mediates the relationship between Perceived Usefulness and Behavioural Intention to Use.	Supported
H5b	Attitude towards Use positively mediates the relationship between Perceived Ease of Use and Behavioural Intention to Use.	Not Supported
H5c	Attitude towards Use positively mediates the relationship between Organisational Support and Behavioural Intention to Use.	Not Supported
H5d	Attitude towards Use positively mediates the relationship between Training and Behavioural Intention to Use.	Supported

## Data Availability

The anonymous data from this study will be used for academic research only. The raw data are not publicly accessible due to ethical and privacy constraints. However, they are available upon reasonable request and with approval from the Multimedia University Research Ethics Committee.

## Abbreviations

The following abbreviations are used in this manuscript:

ED	Emergency department
EDs	Emergency departments
WHO	World Health Organization
TAM	Technology Acceptance Model
TRA	Theory of Reasoned Action
UTAUT	Unified Theory of Acceptance and Use of Technology
PEOU	Perceived Ease of Use
PU	Perceived Usefulness
OS	Organisational Support
T	Training
ATU	Attitude Towards Use
BIU	Behavioural Intention to Use
FC	Facilitating Conditions
EHR	Electronic Health Records
SPSS	Statistical Package for the Social Sciences
SEM	Structural Equation Modelling
PLS-SEM	Partial Least Squares SEM
CMV	Common Method Variance
CMB	Common Method Bias
RMSE	Root-Mean-Square Error
MAE	Mean Absolute Error
IPMA	Importance–Performance Map Analysis
